# Characterization of the N‐Hydroxylating Monooxygenase TheA from Thermocrispum agreste Reveals a Broad Substrate Spectrum

**DOI:** 10.1002/cbic.202500574

**Published:** 2025-09-11

**Authors:** Artur Maier, Daniel Fast, Dmytro Sakalo, Lindelo Mguni, Dirk Tischler

**Affiliations:** ^1^ Faculty of Biology and Biotechnology Microbial Biotechnology Ruhr University Bochum Universitätsstrasse 150 Bochum 44780 Germany

**Keywords:** amino acid biotransformation, biocatalysis, enzyme cascade, flavoprotein monooxygenase, N‐hydroxylation

## Abstract

The *N*‐hydroxylating monooxygenase (NMO) TheA from *Thermocrispum agreste* catalyzes the *N*‐hydroxylation step of l‐ornithine, which is the first step in the thermochelin siderophore biosynthesis. Characterization of this enzyme revealed a significant thermostability up to 50 °C and activity with the non‐native substrate d‐ornithine with kinetic parameters (*K*_m_ = 4.06 ± 0.31 mM, *k*_cat_ = 0.057 ± 0.001 s^−1^, and *k*_cat_/*K*_m_ = 0.007 s^−1 ^mM^−1^) and a coupling rate of 81%. The enzyme is applied in a one‐pot reaction with a formate dehydrogenase variant for NADPH regeneration and catalase for H_2_O_2_ detoxification. Optimization of the reaction conditions resulted in activity with various non‐native substrates such as d‐ornithine, l‐lysine, S‐(2‐aminoethyl)‐l‐cysteine, and l‐arginine. Products are confirmed through LC‐MS/MS, and mutagenesis experiments gave insight on the potentially underlying mechanisms. This work identifies a thermotolerant NMO that is suitable for application and as a starting point for enzyme engineering.

## Graphical abstract

TheA is a moderate thermostable flavin‐dependent monooxygenase which accepts, besides l‐ornithine a number of related molecules for the regio‐selective *N*‐hydroxylation. The combination with a NADP^+^‐reducing formate dehydrogenase variant (FDH M4) and a H_2_O_2_ degrading catalase allows in vitro biocatalytic applications.

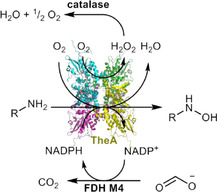

## 1 Introduction

*N*‐hydroxylating monooxygenases (NMOs) are flavin‐dependent enzymes that utilize NADPH as an electron donor to oxidize alkylamines and amino acids, such as ornithine and lysine, to the respective *N*‐hydroxy compounds, under oxygen consumption.^[^ ^1^ ^–^ ^4^ ^]^ These enzymes are found among different bacteria (e.g., Actinomycota, Pseudomonadota) and fungi (e.g., Ascomycota). They catalyze one of the initial steps in secondary metabolite biosynthesis, such as hydroxamate siderophores, piperazate‐containing compounds, and azaserine. Desferrioxamine, a hydroxamate siderophore, is clinically used to treat iron poisoning. Kutznerides, containing the piperazic moiety, and azaserine feature antifungal and antimicrobial properties, respectively.^[^ ^5^ ^]^

**Scheme 1:**
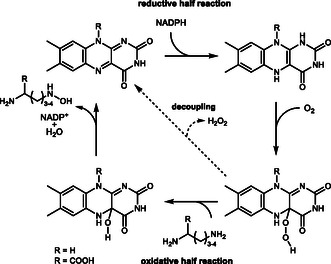
Simplified reaction mechanism of NMOs involving the reductive and oxidative half cycle. After FAD reduction by means of NADPH, the formed NADP^+^ stabilizes the active site and is only released after product hydroxylation. In case of ligand interaction, which cannot be *N*‐hydroxylated, unproductive H_2_O_2_ formation occurs (decoupling).

The reaction cycle consists of a reductive and oxidative half‐reaction. The reductive half reaction involves NADPH binding and subsequent FAD reduction (**Scheme** **1**). NADP^+^ remains bound throughout the catalytic cycle and stabilizes the FAD intermediates. The reductive half reaction is initiated by O_2_ activation by a one‐electron transfer, followed by formation of the C^4a^‐peroxy‐FAD (FAD_OO_^−^), which is protonated to give the C^4a^‐hydroperoxy‐FAD (FAD_OOH_). Upon substrate binding, the intermediate facilitates the hydroxylation by Somersault rearrangement of the hydroxy radical, giving the *N*‐hydroxylated product and the C^4a^‐hydroxy‐FAD (FAD_OH_).^[^ ^6^ ^]^ In the absence of substrate or stabilization, proton transfer from the N^5^ nitrogen of FAD occurs, releasing H_2_O_2_.^[^ ^7^ ^]^ After hydroxylation, FAD reverts to its oxidized state by elimination of H_2_O, which is released with the hydroxylated product and NADP^+^.

The involvement of this enzyme class in a variety of secondary metabolite pathways makes it an interesting target to exploit for the synthesis of useful building blocks, especially as chemical synthesis of *N*‐hydroxy moieties has proved to be challenging due to the difficult control of oxidation states and harsh reaction conditions.^[^ ^8^ ^]^ However, NMOs are known for their rather narrow substrate spectrum, especially the ornithine and lysine NMOs being specific for their respective substrate.^[^ ^4^ ^]^ NMOs accepting diamines were reported to accept putrescine, cadaverine, 1,3‐diaminopropane, spermidine, and lysine.^[^ ^1^ ^–^ ^3^ ^,^ ^9^ ^]^ Here, the enzyme TheA, an NMO from *Thermocrispum agreste*, was characterized, and the substrate scope was further elucidated. It originates from a moderate thermophilic bacterium and thus is stable at elevated temperatures. Moreover, we report on a substrate‐independent low decoupling rate resulting in high efficiency. This low NADPH oxidase activity makes TheA an interesting candidate for biocatalysis.

## 2 Results and Discussion

### 2.1 Alignment and Catalytic Site Analysis

**Figure 1:**
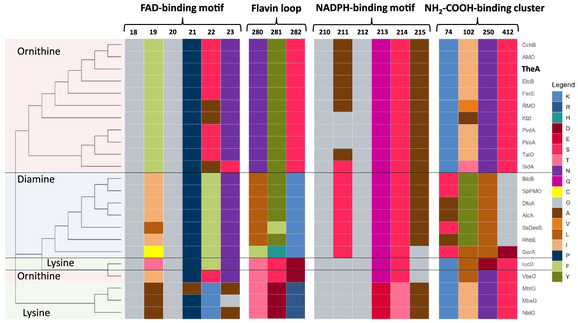
Depiction of the aligned respective binding motifs/binding sites using the A^2^CA tool.^[^ ^35^ ^]^ Shown positions are based on the TheA sequence. Color code indicates the amino acid present in other sequences corresponding to the TheA reference position.

In previous studies, TheA was identified to be part of the thermochelin siderophore biosynthesis cluster, catalyzing the initial *N*‐hydroxylation step, using l‐ornithine as substrate. Sequence alignment of TheA with previously described NMOs (Table S1, Figure S1, Supporting Information) together with phylogenetic analysis (Figure S2, Supporting Information) indicates clearly that TheA belongs to the ornithine hydroxylase family.^[^ ^4^ ^]^ TheA exhibits the typical FAD (GXGXXN), NADPH (GXGQSA) binding motifs as all other NMOs and an amino acid substrate binding site, which is common for *N* ^5^‐l‐ornithine/N^6^‐l‐lysine hydroxylating monooxygenases (**Figure** **1**).^[^ ^6^ ^]^ Moreover, it shares the same FAD loop (^280^NYS^282^), indicating that the FAD undergoes conformational changes upon NADPH binding as reported before for SidA and KtzI.^[^ ^2^ ^,^ ^10^ ^]^ However, diamine and l‐lysine NMOs show more variety in this region, especially the Asn and Ser residues are mostly substituted by Ala and Lys, respectively. Previous studies on SidA showed that substitution of the residues of the flavine loop drastically impacts the kinetic parameters, showing reduced coupling rate and catalytic efficiency,^[^ ^2^ ^]^ which is not the case for the reported diamine NMOs. This raises the question whether they undergo a conformational change at all, as it was not reported yet to the best of our knowledge, or if the underlying mechanism is fundamentally different.

**Figure 2:**
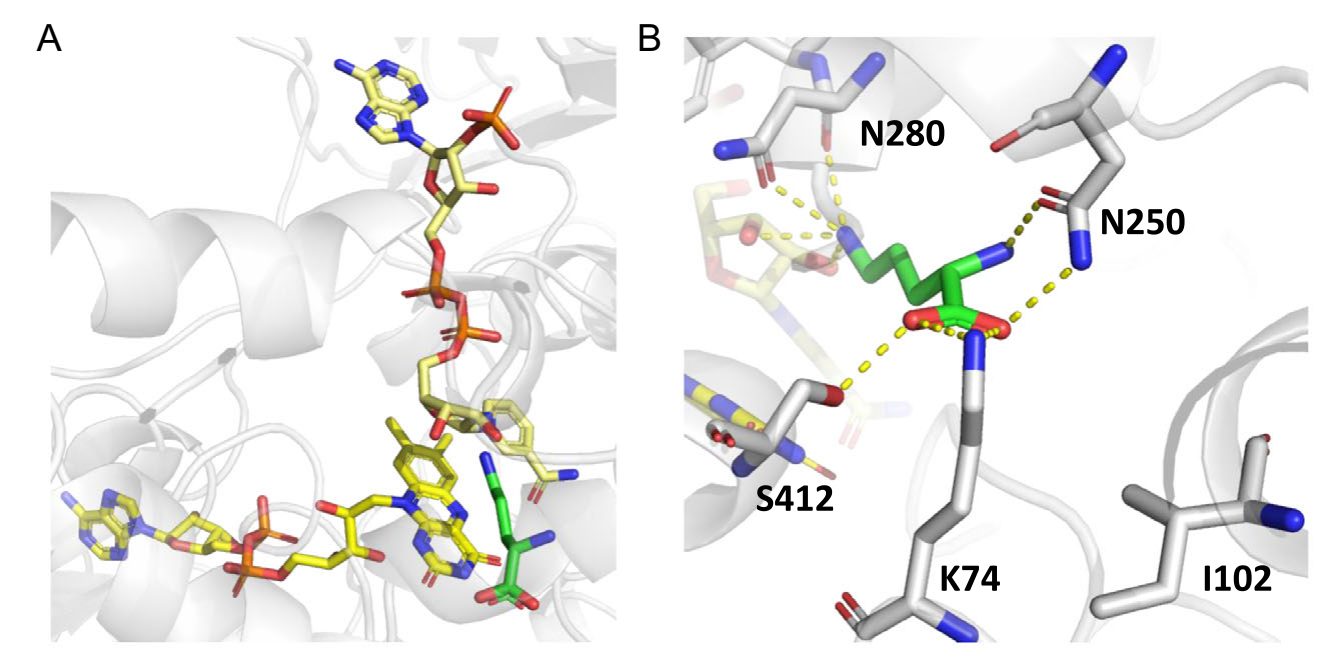
A) FAD, NADP^+^, and l‐Orn orientation in the structure model of TheA. B) Predicted active site residues involved in substrate binding.

Interestingly, the clade consisting of MbtG, MbsG, and NbtG slightly deviates from the conserved FAD and NADPH motifs (Figure 1). However, these motifs are found in the sequence at different positions (FAD motif ^395^GPGFPN^400^ and NADPH motif ^174^GPGQS^178^ for NbtG), especially the FAD motif is located at the *C*‐terminal end of the sequence. Nevertheless, the crystal structure of NbtG with FAD suggests that the above‐reported motifs (Figure 1) are the ones primarily interacting with the respective cofactor, as FAD is co‐crystalized in a similar orientation as in the SidA ref. [2,11]. Since a crystal structure of NbtG with NAD(P)^+^ was not obtained, the binding orientation and the role of the motifs remain to be elucidated. Based on the conserved motifs, we suspected that TheA has many similar characteristics to other known l‐ornithine monooxygenases. The predicted model of TheA suggests a tetrameric form with one active site for each protomer, as was shown for most of the other NMOs. Orientations of FAD and NADP^+^ are similar to those in the crystal structure homologs SidA (**Figure** **2**A).^[^ ^2^ ^]^ Residues K74, N250, and S412 are mainly involved in binding of the amino acid moiety of ornithine and are conserved among the ornithine and lysine accepting NMOs. Among the latter, IucD is the only one with a substitution at N250 to aspartic acid (corresponding to E230 in IucD), likely providing comparable polar bonding capabilities with the *α*‐amino group as asparagine.

### 2.2 Characterization of TheA

TheA was produced with an *N*‐terminal 10x His‐tag. Initial purification procedures following the previously used conditions^[^ ^12^ ^]^ gave low enzyme yields of ≈3.5 mg/L_culture_. As enzyme stability can be influenced by different salt concentrations, modified loading and elution buffers, which contained no additional salts, were tested for the purification.^[^ ^13^ ^]^ With this, yields could be improved by more than twofold up to 1.6 mg/g_wet cells_ or 8.5 mg/L_culture_.

**Figure 3:**
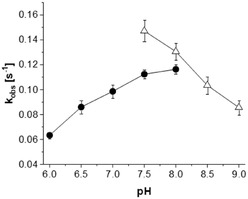
Activities of theA measured by NADPH consumption in dependence on the pH from 6.0 to 9.0, buffered with phosphate buffer (•) and Tris‐HCl buffer (Δ). Measurements were performed with 0.05 mM FAD, 0.15 mM NADPH, and 5 mM l‐Orn. Data were obtained from triplicates, and the standard deviation is indicated by the error bar.

The optima for temperature and pH were measured by means of the NADPH oxidation assay in the presence of substrate. For the measured pH range from 6.0 to 9.0, potassium phosphate buffer (pH 6.0–8.0) and Tris‐HCl buffer (pH 7.5–9.0) were used (**Figure** **3**).

Highest activity was observed in the pH range of 7.5–8.0. This is in contrast to the previously reported homologs CchB and SidA, which prefer a more basic pH range from pH 8.0–9.0.^[^ ^14^ ^,^ ^15^ ^]^ The reason for this difference remains to be elucidated as the amino acid residues involved in the binding of FAD, NADPH, and l‐Orn (pK_a_‐values 1.94, 8.65, and 10.76) are mostly conserved among those NMOs, and the underlying mechanisms for pH preferences is still under debate in this field.^[^ ^15^ ^,^ ^16^ ^]^

**Figure 4:**
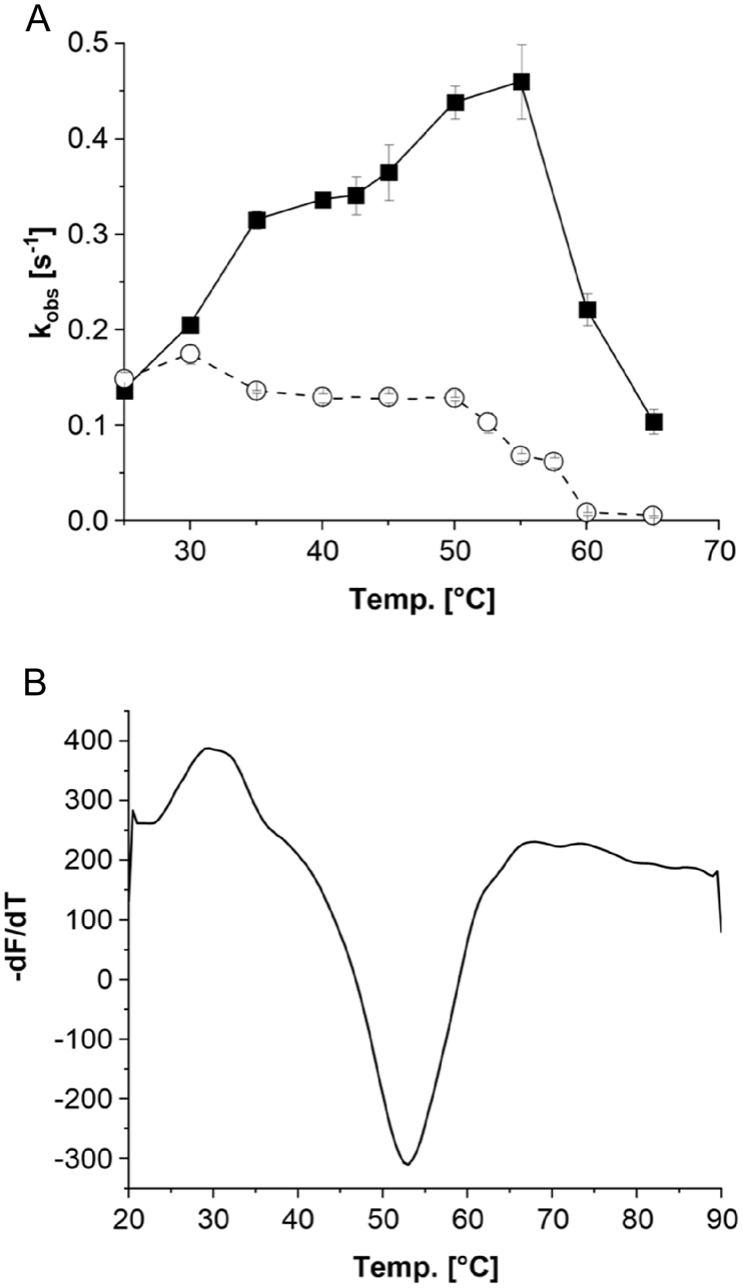
Activities of TheA measured by NADPH consumption. A) Temperature‐dependent activity (▪) and stability (○). For the temperature‐dependent activity, reactions were carried out at the respective temperatures with 2 min preincubation. For the temperature‐dependent stability enzyme was preincubated at the respective temperature for 30 min before activity was determined at 30 °C. Data were obtained from triplicates, and the standard deviation is indicated by the error bar. B) Thermal shift assay. Temperature‐dependent deviation of the fluorescence change. Negative peak indicates the melting point of the enzyme. Data were recorded in 50 mM phosphate buffer (pH 8.0) comprising less than 5% glycerol.

TheA shows the highest activity at 55 °C and remains stable up to 50 °C (**Figure** **4**A), allowing operation at a higher reaction temperature to boost the applicability. This is in agreement with the melting temperature of ≈53 °C determined by the thermal shift assay (Figure 4B) and the conditions in which the host organism *Thermocrispum agreste* is naturally occurring.^[^ ^17^ ^]^ Moreover, TheA contains more cysteines (8) than many other NMOs (Figure S2, Supporting Information), indicating that they may form disulfide bonds for additional stability. However, based on the structure model, many of these cysteines are not close enough to each other to form disulfide bonds (Figure S4, Supporting Information). Only C353 and C372 could potentially form a disulfide bond as C372 is located in a likely more flexible loop region. Either the model needs additional refinement, or most cysteine residues are not involved in disulfide formation and are stabilizing through their hydrophobic properties and inward orientation.

### 2.3 Cofactor Specificity and Cosubstrate Consumption

Heine et al.^[^ ^12^ ^]^ postulated TheA to be FAD and NADPH‐dependent, which is typical for l‐ornithine NMOs. Indeed, the purified enzyme showed a yellow color, suggesting a certain flavin loading. High performance liquid chromatography (HPLC) measurements identified the cofactor as FAD (Figure S5, Supporting Information) with a saturation of ≈20%, which fits in the broad spectrum of NMOs, as homologs were reported to reach cofactor saturations of up to 90% or elute in the apo form.^[^ ^1^ ^,^ ^18^ ^]^ As NMOs in general have a high affinity toward FAD, either the purification conditions are not yet optimized and the FAD is lost upon binding to the His‐Trap column and the subsequent washing steps. Therefore, reconstitution with FAD before measurements was necessary. TheA showed a clear preference for NADPH as a cosubstrate over NADH. The usage of NADH (same concentration as for NADPH) resulted in 70% lower cosubstrate consumption, 80% less product formation, and a twofold increase in peroxide formation (Figure S6, Supporting Information). The reduced affinity of NADH likely results, on the one hand, in less efficient binding of NADH and, on the other hand, in premature dissociation of the NAD^+^, leaving the peroxoflavin intermediate unstabilized, resulting in H_2_O_2_ release. Previous studies identified Arg279 in SidA, a TheA homolog, to be responsible for the NADPH preference.^[^ ^19^ ^]^ Even though TheA contains a Serine (Ser236 in TheA) at the corresponding position according to the alignment (Figure S2, Supporting Information), the next position is Arg237, likely exerting similar interactions. Based on these results, it was decided to focus on NADPH for the following reactions and steady‐state kinetics.

### 2.4 Kinetic Parameters

The steady‐state kinetic parameters were determined using the NADPH oxidation assay and the hydroxylation assay (Figure S7, Supporting Information). The coupling rate was calculated by dividing the *k*_cat_ value for l‐Orn and d‐Orn obtained through the hydroxylation assay by the respective *k*_cat_ value obtained through the NADPH oxidation assay. Results are summarized in **Table** **1**.

**Table 1:** Summary of the kinetic parameters of TheA measured by NADPH oxidation assay and *N*‐hydroxyornithine formation assay. Measurements were conducted with saturation concentrations of NADPH and l‐Orn (for FAD kinetics), FAD and l‐Orn (for NADPH kinetics), and FAD and NADPH (for l‐Orn and d‐Orn kinetics). Data were obtained from triplicates, mean values of initial velocity were plotted to fit the kinetics according to Michaelis–Menten. The standard deviation obtained from the fitting procedure is indicated.

	NADPH oxidation			*N*‐hydroxyornithine formation			
	*K*_m_ [mM]	*k*_cat_ [s^−1^]	*k*_cat_/*K*_m_ [s^−1^ mM^−1^]	*K*_m_ [mM]	*k*_cat_ [s^−1^]	*k*_cat_/*K*_m_ [s^−1^ mM^−1^]	Coupling rate [%]
FAD	1.28 ± 0.43 × 10^−4^	0.13 ± 0.01	1047	–	–	–	–
NADPH (with l‐Orn)	0.04 ± 0.01	0.17 ± 0.01	4.8	–	–	–	–
l‐Orn	0.09 ± 0.01	0.14 ± 0.01	1.5	0.13 ± 0.01	0.11 ± 0.01	0.81	81 ± 3
d‐Orn	3.92 ± 0.27	0.07 ± 0.01	0.02	4.06 ±0.31	0.06 ± 0.01	0.01	81 ± 6

TheA has *K*_m_ values for FAD and NADPH of 1.28 ± 0.43 × 10^−4^ mM and 0.036 ± 0.001 mM, respectively, showing the high affinity for both cofactors. Interestingly, the *K*_m_ value for NADPH reported for the ornithine monooxygenase from *Amycolatopsis alba* (AMO) is ≈72‐fold higher than for TheA. The authors hypothesized that the lower affinity toward NADPH might be due to the absence of the crucial arginine residue (Ala238 in AMO), which is involved in the bonding with the phosphate group of NADPH, as it was shown for SidA.^[^ ^19^ ^,^ ^20^ ^]^ However, the following position R239 (R237 in TheA) might compensate for the missing residue, as in TheA this arginine residue is also shifted by 1 position (Figure S2, Supporting Information). It remains to be elucidated if the presence of the serine (S236 in TheA and A238 in AMO) is probably responsible for the higher affinity due to its hydrogen bonding ability or if other factors are involved. Surprisingly, we observed in preliminary measurements *N*‐hydroxylating activity of TheA with d‐ornithine (d‐Orn) (Figure S7A, Supporting Information) and thus, decided to determine the kinetic parameters alongside l‐Orn. For l‐Orn, TheA shows a relatively low *K*_m_ of 0.134 ± 0.002 mM and a *k*_cat_ of 0.11 ± 0.003 s^−1^, both values fitting well into the variety of kinetic parameters reported for other NMOs.^[^ ^2^ ^,^ ^14^ ^,^ ^21^ ^–^ ^24^ ^]^ The *K*_m_ was 30‐fold higher with d‐Orn, whilst the *k*_cat_ was around 50% lower compared to l‐Orn. Interestingly, the coupling rate under saturation conditions was 81% for both substrates, even though d‐Orn was only known as an effector increasing decoupling rates in NMOs and not being a fitting substrate so far.^[^ ^14^ ^,^ ^21^ ^,^ ^22^ ^]^ This leads to the suspicion that TheA might have a broader substrate spectrum than anticipated.

### 2.5 Reaction Engineering for Substrate Spectrum Screening

To maximize the yield in order to reach the detection range of potential *N*‐hydroxy products when screening substrates, higher NADPH concentrations might be necessary, as it is not unlikely to have higher decoupling rates for alternative substrates. Therefore, we elucidated how higher NADPH concentrations affect the *N* ^5^‐hydroxy‐l‐ornithine (OH‐Orn) and H_2_O_2_ production.

**Figure 5:**
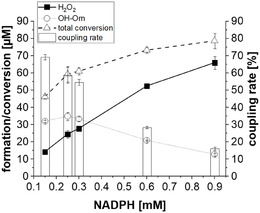
Total NADPH conversion (Δ), H_2_O_2_ (▪), and OH‐Orn (○) formation after 10 min, dependent on the initial NADPH concentration. Reactions were performed in the presence of 5 mM l‐Orn. Coupling rate is shown as bars. Data were obtained from three independent reactions. The mean value is presented, and the standard deviation is indicated by the error bar.

With respect to the unproductive use of NAD(P)H, flavoprotein monooxygenases either follow the so‐called “cautious” or “bold” mechanism to prevent decoupling (NAD(P)H oxidase activity).^[^ ^25^ ^]^ The “cautious” monooxygenases hardly stabilize the reactive peroxoflavin intermediate but rather rely on a fast substrate hydroxylation to outcompete NAD(P)H oxidase activity. On the other hand, the “bold” ones rapidly reduce the flavin cofactor and form the intermediate no matter if substrate is present or not. But they have evolved routes to stabilize the reactive peroxy‐species to lower the decoupling. As proposed for class B FMOs, which follow the “bold” mechanism, the cofactor (here NADP^+^) remains bound after the hydride transfer to stabilize the C^4^‐peroxoflavin intermediate.^[^ ^25^ ^]^ The disruption of the cofactor binding before the substrate hydroxylation takes place results in H_2_O_2_ release, the decoupling. This is also backed up by the results showing higher H_2_O_2_ production and lower *N*‐hydroxy production with increasing NADPH concentration (**Figure** **5**), suggesting that additional NADPH is disrupting the complex. This is in agreement with previous results, where substrate inhibition was observed for SidA if measured by means of the product formation assay.^[^ ^18^ ^]^

**Figure 6:**
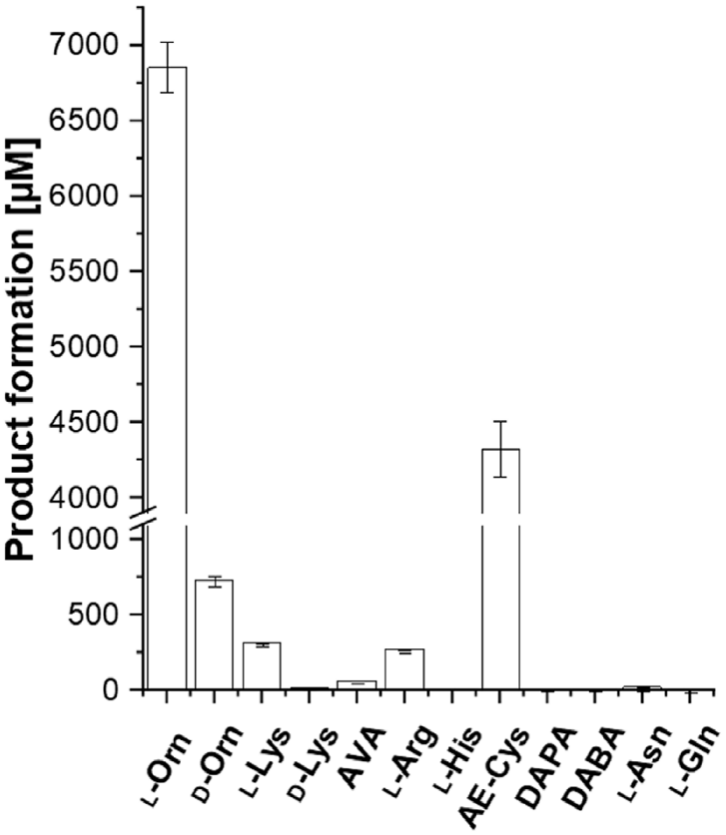
*N*‐hydroxy moiety formation catalyzed by TheA in a one‐pot reaction with FDH M4 and catalase using different substrates (10 mM). The reaction was running at 30 °C for 16 h to prior LC‐MS/MS analytics as described in the methods section. Data were obtained from triplicates (mean value is given), and the standard deviation is indicated by the error bar.

**Scheme 2:**
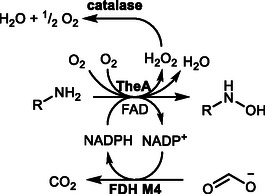
Cascade set up for TheA substrate screening involving FDH M4 for NADPH regeneration,^[^ ^26^ ^]^ catalase for H_2_O_2_ detoxification, and TheA.

**Scheme 3:**
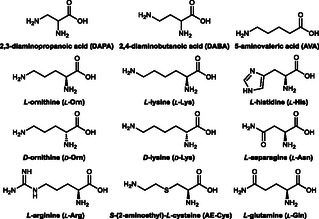
Substrates used for the screening in the cascade setup.

These findings point out that for a reliable screening, the two main aspects, NADPH regeneration and H_2_O_2_ detoxification, have to be addressed, as the potential activity of NMOs with alternative substrates might be overshadowed by high decoupling rates, resulting in fast depletion of NADPH and generation of detrimental H_2_O_2_. With this, constant regeneration of NADPH would be necessary, as higher concentrations would further increase decoupling. To avoid these circumstances, an enzyme cascade was set up involving TheA, the formate dehydrogenase variant C23S/D195Q/Y196R/Q197N (FDH M4) from *Candida boidinii* as a NADPH regeneration system to guarantee a constant supply of NADPH and catalase for the removal of accumulating H_2_O_2_ (**Scheme** **2**). With this setup various alternative substrates were screened for *N*‐hydroxy compound formation (**Scheme** **3**, **Figure** **6**).^[^ ^26^ ^]^

**Figure 7:**
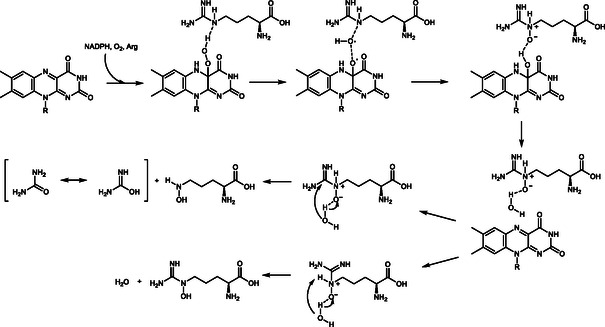
Putative reaction mechanism of TheA with arginine based on previous studies.^[^ ^28^ ^]^ The water released from FAD protonates the hydroxylamine oxygen and subsequently either deprotonates the nitrogen or attacks at the carbon, yielding *N*‐hydroxyarginine and water or *N*‐hydroxyornithine and urea.

TheA showed some promiscuity toward some alternative substrates such as d‐Orn, l‐Lys, AE‐Cys, Arg, and AVA, giving a positive signal for *N*‐hydroxy compound formation (Figure 6). Remarkably, we could detect *N*‐hydroxy compound formation with l‐Lys as substrate, even though in our first measurements (Figure S5, Supporting Information), only increased H_2_O_2_ production was observed. This shows that a constant supply of NADPH and removal of H_2_O_2_ can reveal activities, not detected in the first place. AE‐Cys seemed to be more acceptable than l‐Lys despite having a similar chain length. To further analyze the products, LC‐MS/MS measurements were conducted. For reactions with l‐Orn, the respective OH‐l‐Orn with a m/z (+) value of 149.09 was detected and subsequently fragmented by collision‐induced dissociation (CID). The obtained mass spectrum was analyzed based on previous studies.^[^ ^27^ ^]^ All detected fragments could be associated with OH‐l‐Orn (Figure S8, Supporting Information). Similar results were obtained for d‐Orn reactions (Figure S9A, Supporting Information). It needs to be mentioned that our chromatographic setup resulted in a major product peak at 4.3 min for OH‐l‐Orn and at 5.1 min for OH‐d‐Orn, respectively, as well as minor product peaks at inverted retention times (Figure S8 and S9, Supporting Information). Both of these peaks show the same mass spectrum and fragmentation pattern. Thus, in both cases, while producing mainly one OH‐Orn enantiomer, a peak of the other enantiomer was observed, which is likely due to the applied substrate (purity of 99% and 98%, respectively) and the biotransformation setup. For l‐Lys and AE‐Cys reactions, the putative *N*‐hydroxy products (OH‐Lys and OH‐AE‐Cys) with m/z (+) values of 163.11 and 181.06 were detected. After fragmentation of the precursors and analysis of the obtained mass spectrum, all fragments could be associated with the respective *N*‐hydroxy products (Figure S10–S11, Supporting Information). No products were detected for reactions with 5‐aminovaleric acid (AVA). However, this might be due to the low amount of *N*‐hydroxy product produced and the absence of the second primary amine group, hampering ionization as the measurements were conducted in positive mode. Interestingly, LC‐MS analysis of the Arg reaction revealed formation of OH‐l‐Orn and the putative OH‐Arg (Figure S12A, Supporting Information). Fragmentation of the putative OH‐l‐Orn peak gave the same fragments as detected before for OH‐l‐Orn (Figure S8 and Figure S12B,C, Supporting Information), further suggesting the formation of the *N*‐hydroxy product. For OH‐Arg, many fragments were detected, and the four most prominent could be associated with the proposed fragmentation pathway (Figure S12D–F, Supporting Information), indicating that OH‐Arg might undergo divergent fragmentation due to the altered chemical properties. Based on the proposed reaction mechanism, it can be hypothesized that two products can be formed. But which of those is generated might be directed by the water‐catalyzed protonation/deprotonation step.^[^ ^28^ ^]^ Either the attack happens at the hydrogen giving OH‐Arg or at the carbon of the guanidinium moiety, resulting in hydrolysis into OH‐Orn and urea (**Figure** **7**). However, the exact mechanism still has to be revealed.

Compared to other NMOs, TheA seems to have a broader substrate spectrum as compared to other ornithine NMOs such as CchB, VbsO, SidA, and KtzI, which only accept l‐Orn as substrate and showed partially increased decoupling with l‐Lys and d‐orn.^[^ ^14^ ^,^ ^18^ ^,^ ^21^ ^,^ ^22^ ^]^ The lysine NMO NbtG shows activity with l‐ and d‐Lys with a clear preference toward l‐Lys, as the K_m_ for d‐Lys is more than sevenfold higher, whereas IucD was specific for l‐Lys.^[^ ^11^ ^,^ ^29^ ^]^ This resembles the observed behavior of TheA with l‐ and d‐orn, even though the K_m_ for d‐Orn is significantly higher. However, in contrast to NbtG, TheA has a rather low decoupling rate of <20%, making it more applicable in the context of biocatalysis.

### 2.6 Mutagenesis of the Substrate Binding Site

**Figure 8:**
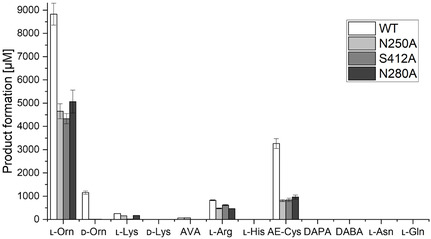
*N*‐hydroxy moiety formation catalyzed by TheA variants N250A, S412A, and N280A in a one‐pot reaction with FDH M4 and catalase using different substrates. Data were obtained from triplicates, and the standard deviation is indicated by the error bar.

To get further insight into the role of the conserved residues likely responsible for amino acid substrate binding (Figure 2B), mutagenesis studies were conducted. Variant K74A could not be produced (Figure S14, Supporting Information), indicating its structural importance. All mutations (N250A, N280A, and S412A) resulted in a significant reduction in product formation of up to 50% (**Figure** **8**). Interestingly, every mutation completely aborted hydroxylation of d‐Orn, suggesting that the interplay of all residues is crucial for its utilization. Whereas for l‐Arg, the overall loss was less prominent. Moreover, it shows that bigger active sites do not necessarily improve the acceptance of bigger substrates, further underlining the importance of ideal orientation for the reaction to take place. Remarkably, despite being part of the predicted NYS flavin loop and thus its proximity to FAD and the terminal amine group of the substrate (Figure 1 and 2B), variant N280A showed distinct product formation. On the one hand, this may partly explain the loss of activity; on the other hand, it raises the question of whether Gln plays a crucial role in flavin dynamics as initially predicted. Moreover, it is predicted to be involved in interactions with the N^5^‐atom of l‐Orn. Mutation of E216 in the homolog NbtG, which forms H‐bonds with the N^6^‐atom of l‐Lys, distinctly impaired l‐Lys hydroxylation.^[^ ^30^ ^]^ Overall, these results underline the involvement of the residues N250, N280, and S412 in substrate acceptance as shown for other NMOs such as SidA or PvdA, among others.^[^ ^2^ ^,^ ^31^ ^]^

## 3 Conclusion

The *N*‐hydroxylating monooxygenase TheA from *Thermocrispum agreste* showed increased thermostability up to 50 °C and decent activity with l‐ornithine, with *K*_m_ and *k*_cat_ values of 0.134 ± 0.002 mM and 0.11 ± 0.003 s^−1^. Due to the thermostability, the activity could potentially be boosted by increasing the reaction temperature and by longer reaction times, which is not possible for other NMOs due to their low thermostability.^[^ ^1^ ^]^ With reaction engineering, it was unveiled that TheA has a broader substrate spectrum than most of the reported NMOs so far.^[^ ^1^ ^,^ ^2^ ^,^ ^14^ ^,^ ^21^ ^–^ ^23^ ^]^ This was achieved by utilizing a formate dehydrogenase variant for NADPH regeneration and catalase for H_2_O_2_ detoxification.^[^ ^26^ ^]^ These results put the already reported substrate specificities into perspective, as it is possible that activities were overseen due to the high decoupling rates and that, if the decoupling and H_2_O_2_ are taken out of the equation, NMOs might have a broader substrate spectrum than previously anticipated. It is important to note that reactions with the non‐native substrates are still uneconomical and require further optimization to be suited for application. However, this opens new possibilities such as enzyme engineering, which could enable the application of NMOs as biocatalysts.

## 4 Experimental Section

4.1

4.1.1

##### General Experimental Methods

Chemicals and reagents were purchased at the highest purity from Carl–Roth, TCI, and Sigma–Aldrich. They were used without further purification. The expression vector pET16bP was used for protein production. All constructs were initially transformed to *Escherichia coli* DH5, then the plasmids were isolated using the NucleoSpin plasmid mini prep kit for plasmid DNA (Macherey–Nagel). Preculture for expression strains was done using Lysogeny broth (LB) with 10 g L^–1^ of tryptone, 10 g L^–^^1^ of NaCl, and 5 g L^–^^1^ of yeast extract. A final concentration of 100 μg mL^–^^1^ ampicillin was used for LB broth and agar plates. Cultures were incubated at 37 °C for 24 h, 140 rpm.

##### Bioinformatic Binding Site Analysis

An alignment of TheA and homologs (Table S1, Supporting Information) was generated using MEGA11 software^[^ ^32^ ^]^ and the MUSCLE algorithm with the general settings.^[^ ^32^ ^,^ ^33^ ^]^ Results were visualized using ESPript, and conserved residues are highlighted in red (Figure S2, Supporting Information).^[^ ^34^ ^]^ A phylogenetic tree of TheA and homologs was created using the minimum evolution method. Both datasets were combined with the A^2^CA^[^ ^35^ ^]^ tool in order to highlight residues of interest in the phylogenetic context.

##### Site‐Directed Mutagenesis

All mutagenesis reactions were performed using the QuiK‐Change method following manufacturer instructions. The *taTheA* containing the pET16bp construct was used as a template.^[^ ^12^ ^]^ The mutagenesis primers used were designed using SnapGene, ensuring that the desired mutation was centrally within the primer sequence (see Table S2, Supporting Information). Specific nucleotide changes were done at N280, N250, S412, and K74, replacing each with an alanine (A). The reaction mixture for PCR contained 12.5 µL of PrimeSTAR Max Mastermix (2 mM Mg^2+^, 0.4 mM each dNTP), 1 µL of each primer (10 µM), 1 µL plasmid DNA, and nuclease‐free water added to a total of 25 µL. The PCR reaction was conducted as follows: initial denaturation at 98 °C, 120 s; denaturation at 98 °C, 20 s; annealing at 68 °C, 20 s; elongation at 72 °C, 120 s; and final elongation at 72 °C, 300 s. A total of 30 cycles were done, followed by DPN1 digest (37 °C, 1 h) to remove the template plasmid DNA. After digestion, the mixture was used to transform into *E. coli* DH5*α* cells and inoculated onto LB agar medium (100 µg mL^–^^1^ ampicillin) and incubated (37 °C, 16 h). Three colonies of each mutation were picked for plasmid extraction. Plasmids were sent for Sanger sequencing at Microsynth Seqlab GmbH to determine and verify the insertion of the necessary mutations.

##### Expression and Purification

The *E. coli* NiCo21 (DE3) cells were transformed by a previously generated *taTheA* containing pET16bp construct,^[^ ^12^ ^]^ or the plasmid harboring the introduced mutations. Expression cultures were cultivated at 37 °C in Lysogeny broth (LB) medium in baffled flasks and 400 rpm until OD_600_ reached ≈0.4. Afterward, cultures were cooled down to 20 °C and incubated for 1 h after the temperature change to ensure a complete cool down. Finally, cultures were induced with 1 mM isopropyl *β*‐d‐1‐thiogalactopyranoside (IPTG) and incubated for at least 16 h at 20 °C to avoid formation of inclusion bodies. Cells were harvested, resuspended in loading buffer (50 mM potassium phosphate buffer, pH 8.0, and 30 mM imidazole), and disrupted through sonication (30% intensity, 30 s on and 60 s break, constantly on ice for 5 cycles). Soluble fractions were obtained by centrifugation and applied to a 1 mL HisTrap column. The enzyme was eluted with 100% elution buffer (50 mM potassium phosphate buffer, pH 8.0, and 500 mM imidazole). Elution fractions were dialyzed against 50 mM phosphate buffer, pH 8.0, and stored in storage buffer (50 mM potassium phosphate buffer, pH 8.0, and 50% (v/v) glycerol) at –20 °C. Protein concentrations were determined using the Bradford method.^[^ ^36^ ^]^

##### Thermal Shift Assay

Thermal shift assay was performed with SYPRO orange dye. Enzyme stocks were diluted with 50 mM phosphate buffer to 5 µM (pH 8.0). 20 µl of diluted enzyme was transferred into qPCR‐tubes and 5 µl of 5x SYPRO orange dye was added. The qPCR device method was set to 20–90 °C with an increment of 0.5 °C for 10 s.

##### NADPH Oxidation Assay

The standard reaction mixture contained 50 mM phosphate buffer, pH 8.0, 0.05 mM FAD, 0.15 mM NADPH, 5 mM diamino substrate, and 0.05 mg mL^–^^1^ TheA. Reactions were set up in a 1 mL cuvette and preincubated at 30 °C. Reaction was started by the addition of NADPH. Subsequently, absorbance at 340 nm was tracked and served as a measure of activity. For kinetic measurements, concentrations of FAD (up to 0.18 mM), NADPH (up to 0.2 mM), and diamine substrates (up to 25 mM) were used.

##### Hydroxylation Assay

Determination of *N*‐hydroxylated products was performed as described previously.^[^ ^37^ ^]^ Biocatalytic reactions (40 µL per sample) were quenched with 20 µL of 1.5 M perchloric acid and centrifuged at max rpm for 5 min. Subsequently, 40 µL of the supernatant was transferred into 96‐well plates and neutralized with 50 µL of 10% (w/v) sodium acetate solution, followed by the addition of 50 µL of 1% (w/v) sulfanilic acid (in 25% (v/v) acetic acid). Samples were reduced by 20 µL of 1% (w/v) I_2_ (in glacial acetic acid) for 15 min. Excess of I_2_ was removed by the addition of 20 µL of 0.1 M sodium thiosulfate. Finally, 20 µL of 0.6% (w/v) *α*‐naphthylamine (in 30% acetic acid) was added and incubated for 45 min. The formed dye was measured at 562 nm.

##### Hydrogen Peroxide Assay

Formation of the byproduct hydrogen peroxide was measured using the xylenol orange hydrogen peroxide assay.^[^ ^38^ ^]^ 20 µL of standard reactions were transferred onto a new 96‐well plate, already containing 180 μL of detection solution (250 μM FeSO_4_, 25 mM H_2_SO_4_, 100 μM xylenol orange). In the presence of hydrogen peroxide, the absorption of the dye shifts from 440 to 560 nm, which was measured after incubation for 30 min.

##### Cofactor Identification by HPLC

Measurements were conducted using a Thermo UltiMate 3000 HPLC. LC separation of the analytes was achieved using a Knauer Eurosphere 100–5 C18 (125 × 4 mm) column with the mobile phase A consisting of H_2_O with 50 mM ammonium acetate, pH 5.0, and mobile phase B consisting of methanol. Elution program was a linear gradient (30–60% B within 8 min, with initial 2 min at 30% B) with a flow rate of 0.7 mL min^–^^1^. TheA samples were denatured by incubating at 90 °C for 10 min, and the supernatant was used for measurements.

##### One‐Pot Reaction for Substrate Screening

Cascade reactions contained 50 mM phosphate buffer, pH 8.0, 100 mM sodium formate, 0.1 mM FAD, 0.2 mM NADPH, 0.12 mg mL^–^^1^ (2.8 µM) FDH M4,^[^ ^26^ ^]^ 0.04 mg mL^–^^1^ (0.7 µM) catalase (bovine liver, Sigma), 0.2 mg mL^–^^1^ (4 µM) TheA, and 10 mM of the respective substrate. Reactions were incubated at 30 °C for 16 h. Afterward, measurements were conducted according to the respective assays.

##### LC‐MS/MS Analysis

Measurements were conducted using the UHPLC Nexera X2 device coupled with the Shimadzu LCMS 8030 mass spectrometer. LC separation of the analytes was achieved through an isocratic HILIC method (70% Acetonitrile/30% H_2_O + 20 mM ammonium formate buffer, pH 6.0, flow rate of 0.5 mL min^–1^) using an Ascentis bare silica HILIC column (2.7 µm 3 mm ×15 cm). Substrates and corresponding *N*‐hydroxylated products were detected by single ion monitoring (SIM) in positive mode utilizing an electron spray ionization (ESI) unit. The respective [M+H]^+^ ions were detected and identified by subsequent CID with argon gas and a collision energy of 20 V. Mass spectra of the respective analytes were extracted from the product ion scans of the [M+H]^+^ ions. Fragmentation patterns were predicted based on previous studies.^[^ ^27^ ^]^

##### Statistical Analysis

In general, all enzyme activity data reported based on at least three independent measurements (triplicates). Therefore, the enzyme from the same purification batch was quantified and applied to the respective analysis. Data obtained were analyzed and quantified as described in the assay section and directly used to calculate rates or yields accordingly. The data obtained were fitted using OriginPro 2019. If not otherwise stated, data are presented as mean value ± standard deviation (either in text or as a bar in figures).

## Supporting Information

The authors have cited additional references within the Supporting Information.^[^ ^39^ ^]^

## Conflict of Interest

The authors declare no conflict of interest.

## Supporting information

Supplementary Material

## Data Availability

The data that support the findings of this study are available from the corresponding author upon reasonable request.
