# Supplementary material for: Characterization of the N‐Hydroxylating Monooxygenase TheA from Thermocrispum agreste Reveals a Broad Substrate Spectrum
Source: Chembiochem. 2025 Sep 11;26(19):e202500574. doi: 10.1002/cbic.202500574 (PMC12498188; doi:10.1002/cbic.202500574)
Supplement: Supplementary file 1 — Supplementary Material [file CBIC-26-e202500574-s001.pdf]

Supporting Information

Characterization of the *N*-hydroxylating monooxygenase TheA from *Thermocrispum agreste* reveals a broad substrate spectrum

Artur Maier, Daniel Fast, Dmytro Sakalo, Lindelo Mguni, Dirk Tischler*

[a] Dr. A. Maier, D. Fast, Dmytro Sakalo, Lindelo Mguni, Prof. Dr. D. Tischler
Faculty of Biology and Biotechnology
Microbial Biotechnology, Ruhr University Bochum
Universitätsstrasse 150, 44780 Bochum (Germany)
E-mail: dirk.tischler@rub.de

***Table S1**: Reported NMOs*

NMO	Putative Substrate(s)	Organism	Accesion number	reference
**GorA**	Put	*Gordonia rubripertincta*	AOR50757	[1]
**Rmo**	Orn	*Rhodococcus jostii RHA1*	ABG96502	[2]
**DfoA**	Cad	*Erwinia amylovora*	CBA23306	[3]
**MbsG**	Lys	*Mycolicibacterium smegmatis*	WP_011729884.1	[4]
**MbtG**	Lys	*Mycobacterium tuberculosis*	KBJ30699	[5]
**NbtG**	Lys	*Nocardia farcinica*	BAD55606	[6]
**IucD**	Lys	*E. coli*	QBQ68950	[7]
**ktzI**	Orn	*Kutzneria sp. 744*	ABV56589	[8]
**SsDesB**	Cad	*Streptomyces sviceus*	6XBC_H	[9]
**SidA**	Orn	*Aspergillus fumigatus*	AAT84594.1	[10]
**PvdA**	Orn	*Pseudomonas aeruginosa*	AAX16346	[11]
**TheA**	Orn	*Thermocrispum agreste*	PZM95345.1	[12]
**SpPMO**	Put/Lys	*Shewanella putrefaciens 95*	MH899123	[13]
**PsbA**	Orn	*Pseudomonas sp. B10*	AAG27518	[14]
**TaiO**	Orn	*Cupriavidus taiwanensis*	WP_012356056	[15]
**FscE**	Orn	*Thermobifida fusca*	PZN64719	[16]
**EtcB**	Orn	*Saccharopolyspora erythraea NRRL 2338*	PFG94420	[17]
**AMO**	Orn	*Amycolatopsis alba DSM 44262*	OXM54439	[18]
**CchB**	Orn	*Streptomyces ambofaciens ATCC 23877*	A0ACR3	[19]
**VsbO**	Orn	*Rhizobium etli CFN 42*	Q2JYJ0	[20]
**BibB**	Cad	*Aliivibrio salmonicida LFI1238*	CAQ77820.1	[21]
**AlcA**	Put	*Bordetella bronchiseptica RB50*	Q44740	[22]
**RhbE**	Diamino-propane	*Sinorhizobium meliloti 1021*	Q9Z3Q8	[23]

***Table S2**: Quik-Change Mutagenesis Primers*

Primer Name	Sequence (5' to 3')
N280A forward	GGATTATCACGCTAATACAGCTTACTCTGTTGTTGACTTGGATTTAATC
N280A reverse	GATTAAATCCAAGTCAACAACAGAGTAGCTTGTATTAGCGTGATAATCC
N250A forward	CACCAGCTGACGATTCTCCTTTAGCGGCTCGTATTTTTGATCCTGCTACTG
N250A reverse	CAGTAGCAGGATCAAAAATACGGCTCGCTAAAGGAGAATCGTCAGCTGGTG
S412A forward	CATACGCATGGCATCACTTCATCACTATTAGCAAATTGTGCTGTTCGTGC
S412A reverse	GCACGAACAGCACAATTGCATAATAGTGATGAAGTGATGCCATGCGTATG
K74A forward	CTACTATGCAAGTAAGCTTCTTAGCTGATTTGGTAACTTTGCGTAACC
K74A reverse	GGTTACGCAAAGTTACCAAATCGCTTAAGAAGCTTACTTGCATAGTAG

Figure S1: Phylogenetic tree of reported NMOs ... 3
Figure S2: Multiple sequence alignment of reported NMOs ... 5
Figure S3: Alignment of the TheA structure models predicted by AlphaFold and YASARA ... 6
Figure S4: Depiction of the cysteine residues located in the TheA structure model ... 7
Figure S5: TheA Cofactor determination. ... 7
Figure S6: NAD(P)H dependent relative activity of TheA. ... 8
Figure S7: Relative activity of TheA with D-Orn, L-Orn, D-lys and L-Lys ... 8
Figure S8: Kinetic parameters of TheA with varying concentrations of FAD, NADPH, L-Orn and D-Orn. ... 9
Figure S9: LC-MS/MS measurements of TheA cascade biocatalysis with L-Orn ... 10
Figure S10: LC-MS/MS measurements of TheA cascade biocatalysis with D-Orn ... 11
Figure S 11: LC-MS/MS measurements of TheA cascade biocatalysis with L-Lys ... 12
Figure S12: LC-MS/MS measurements of TheA cascade biocatalysis AE-Cys ... 13
Figure S13: LC-MS/MS measurements of TheA cascade biocatalysis with L-Arg ... 14

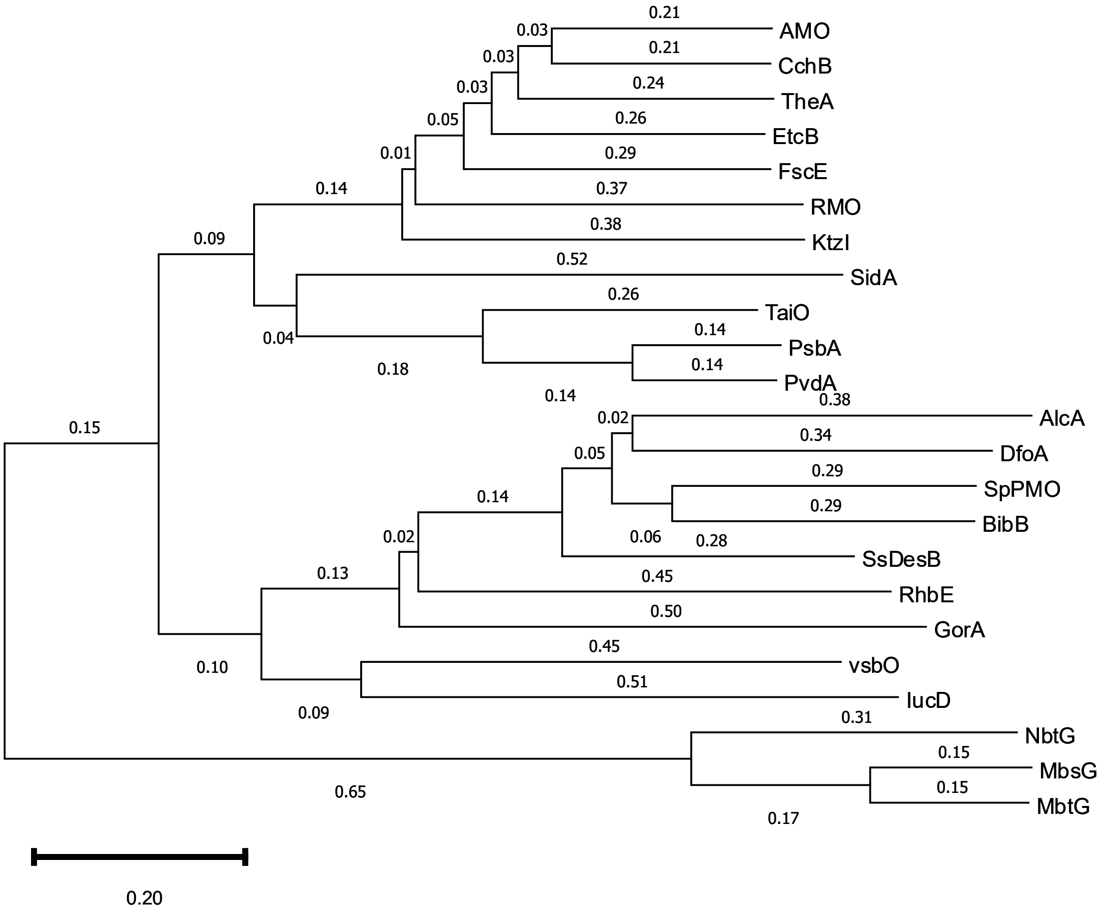

***Figure S1**: Phylogenetic tree of reported NMOs (Table S1) using MEGA11 and the minimum evolution method.*[24]

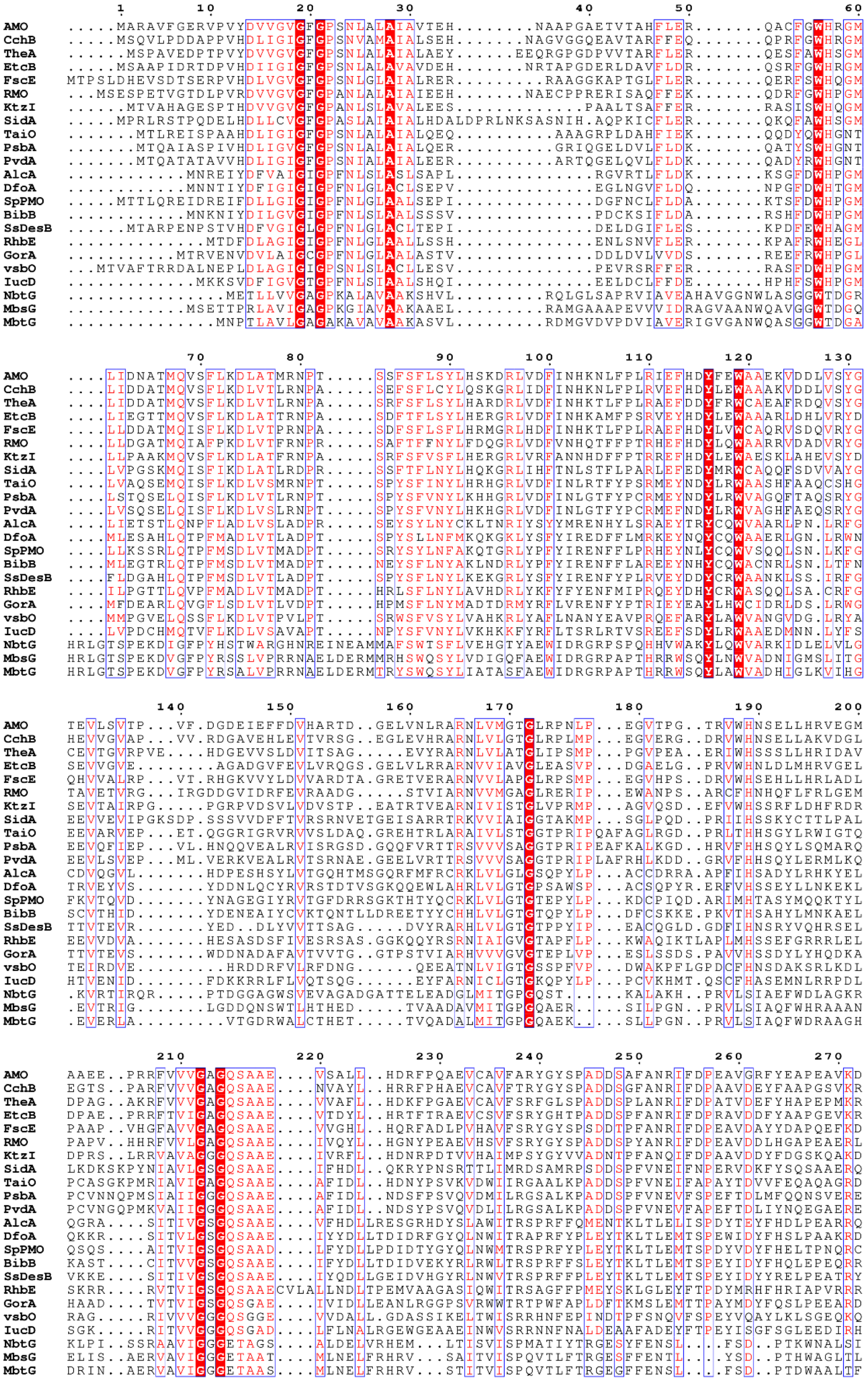
AMO
CchB
TheA
EtcB
FscE
RMO
KtzI
SidA
TaiO
PsbA
PvdA
AlcA
DfoA
SpPMO
BibB
SsDesB
RhbE
GorA
vsbO
IucD
NbtG
MbsG
MbtG

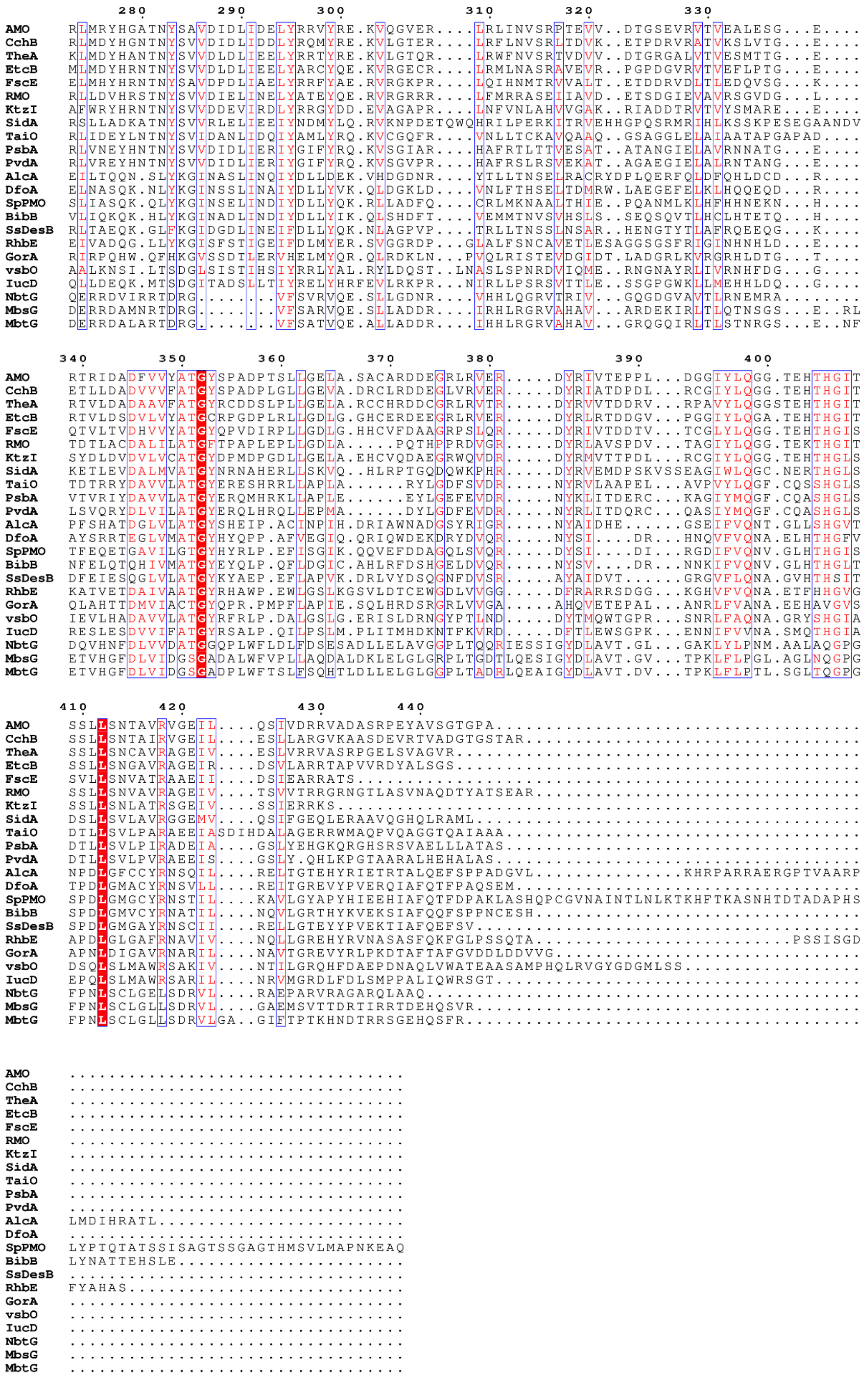

***Figure S2**: Multiple sequence alignment of reported NMOs (Table S1) calculated using MEGA11 and the MUSCLE algorithm.*[24,25]

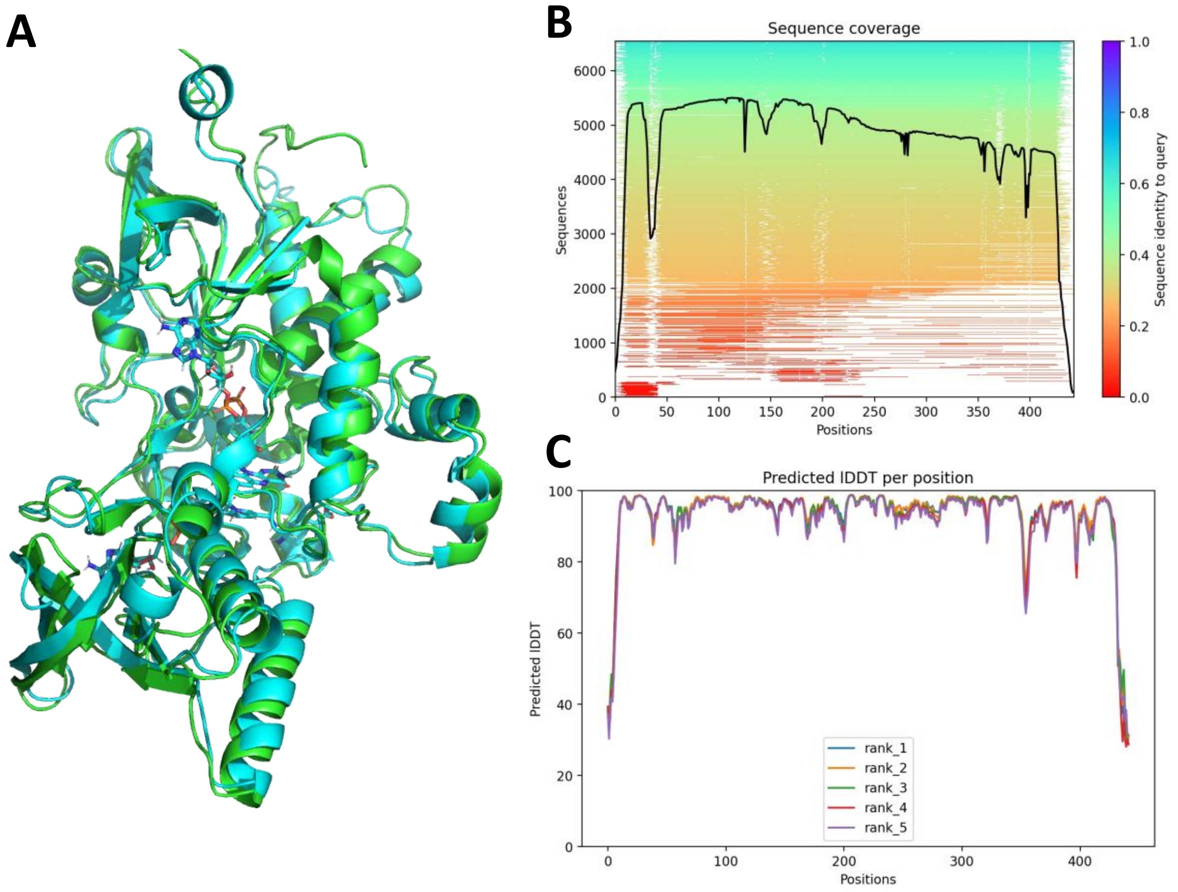

Figure S3: *(A) Alignment of the TheA structure models predicted by AlphaFold (green) and YASARA (cyan). (B) Sequence coverage for TheA structure prediction by AlphaFold. (C) Predicted IDDT for the ranked models predicted by AlphaFold.*[26]

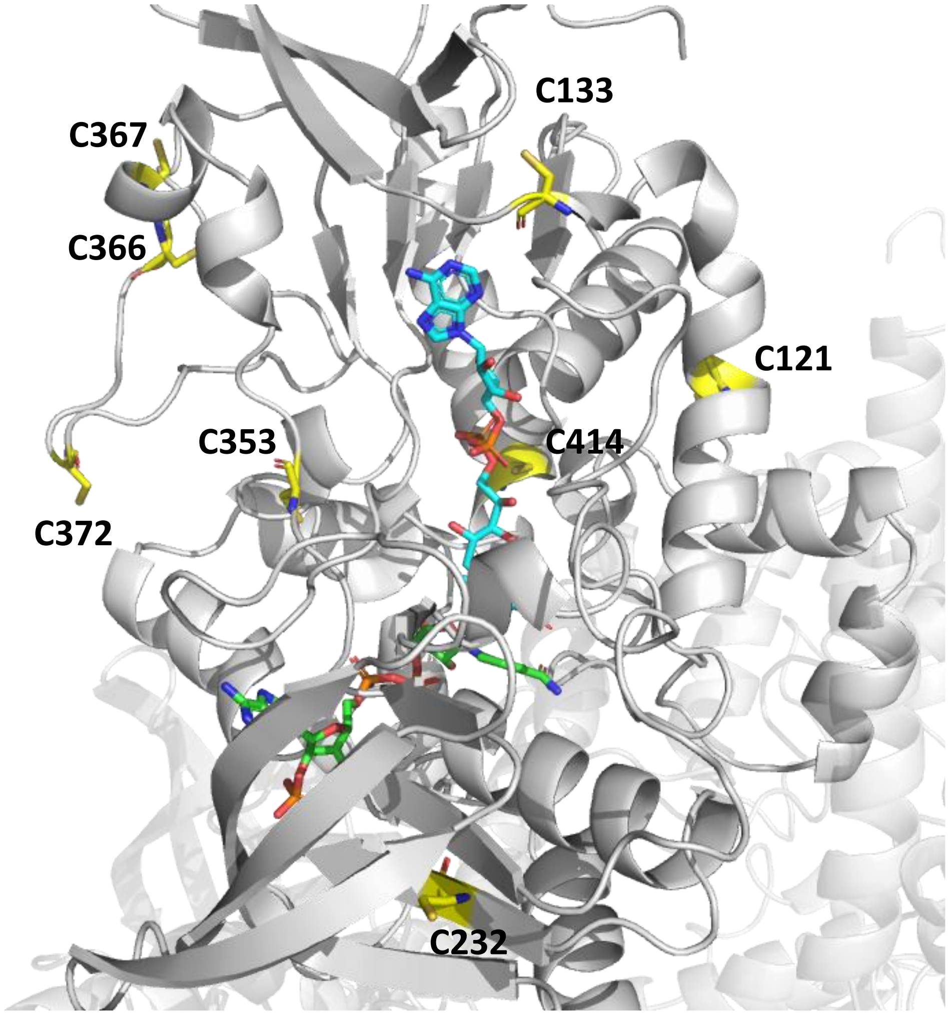

Figure S4: *Depiction of the cysteine residues located in the TheA structure model.*

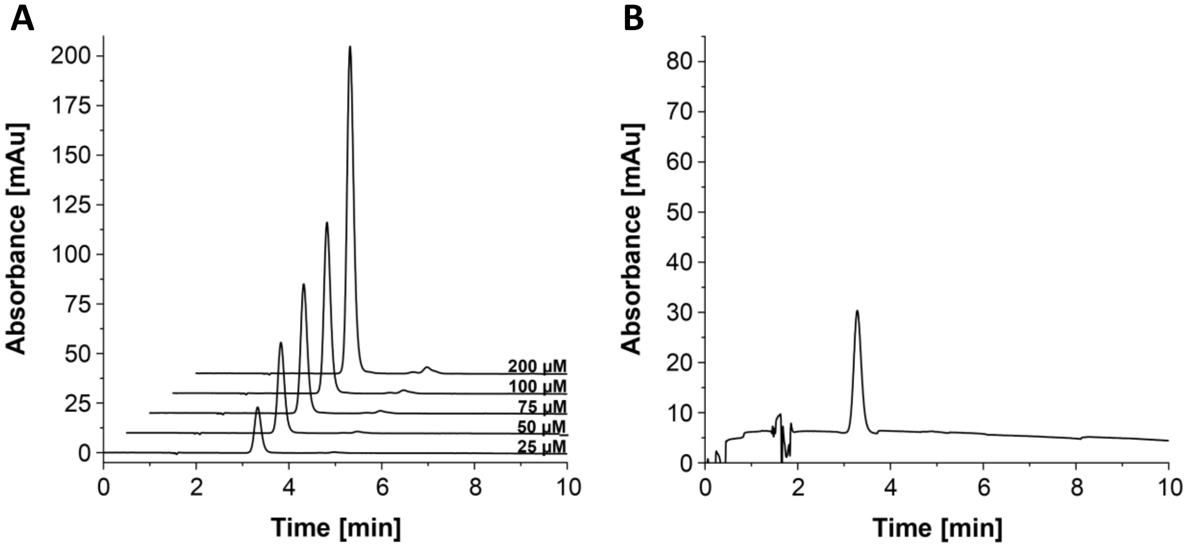

Figure S5: *(A) HPLC-chromatograms of FAD concentrations varying from 25 – 200 µM. Chromatograms are displayed with a time offset of 0.5 min and the absorbance offset of 10 mAu for better visualization. (B) Chromatogram of the free cofactor after denaturation of the purified TheA.*

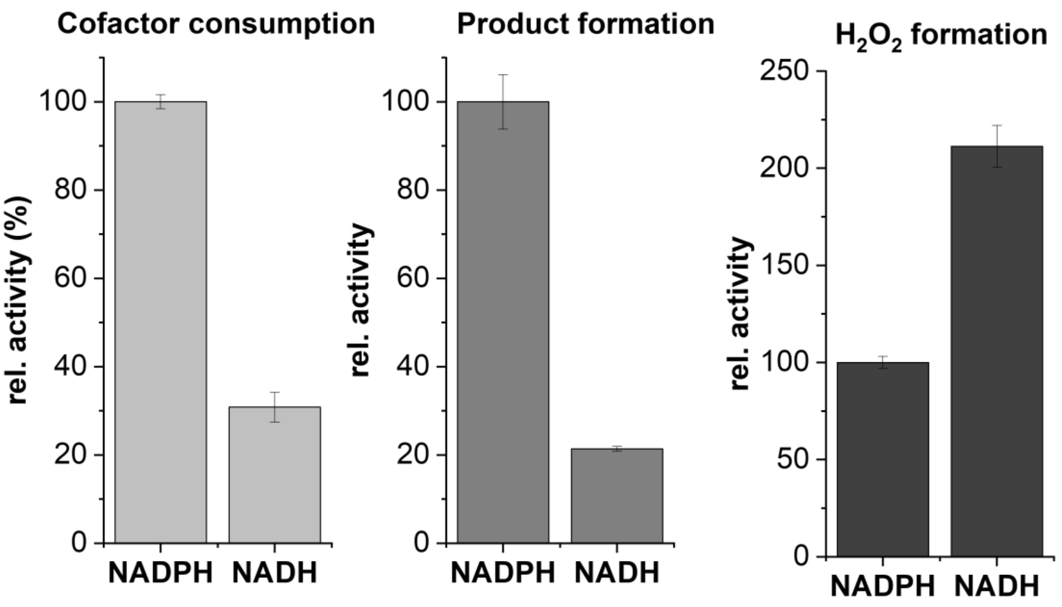

Figure S6: *NAD(P)H dependent relative activity of TheA based on cofactor consumption (NAD(P)H oxidation assay), product formation (Hydroxylation assay), and H_2O_2 formation (Xylenol-orange assay). NADPH and NADH concentrations were 0.15 mM.*

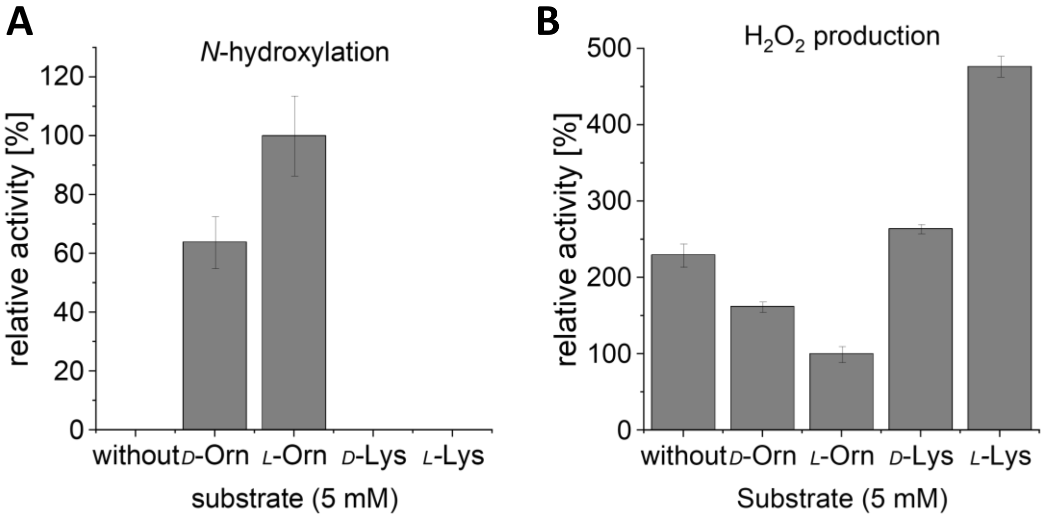

Figure S7: *Relative activity of TheA with D-Orn, L-Orn, D-lys and L-Lys by means of the (A) hydroxylation assay and (B) H_2O_2 production assay.*

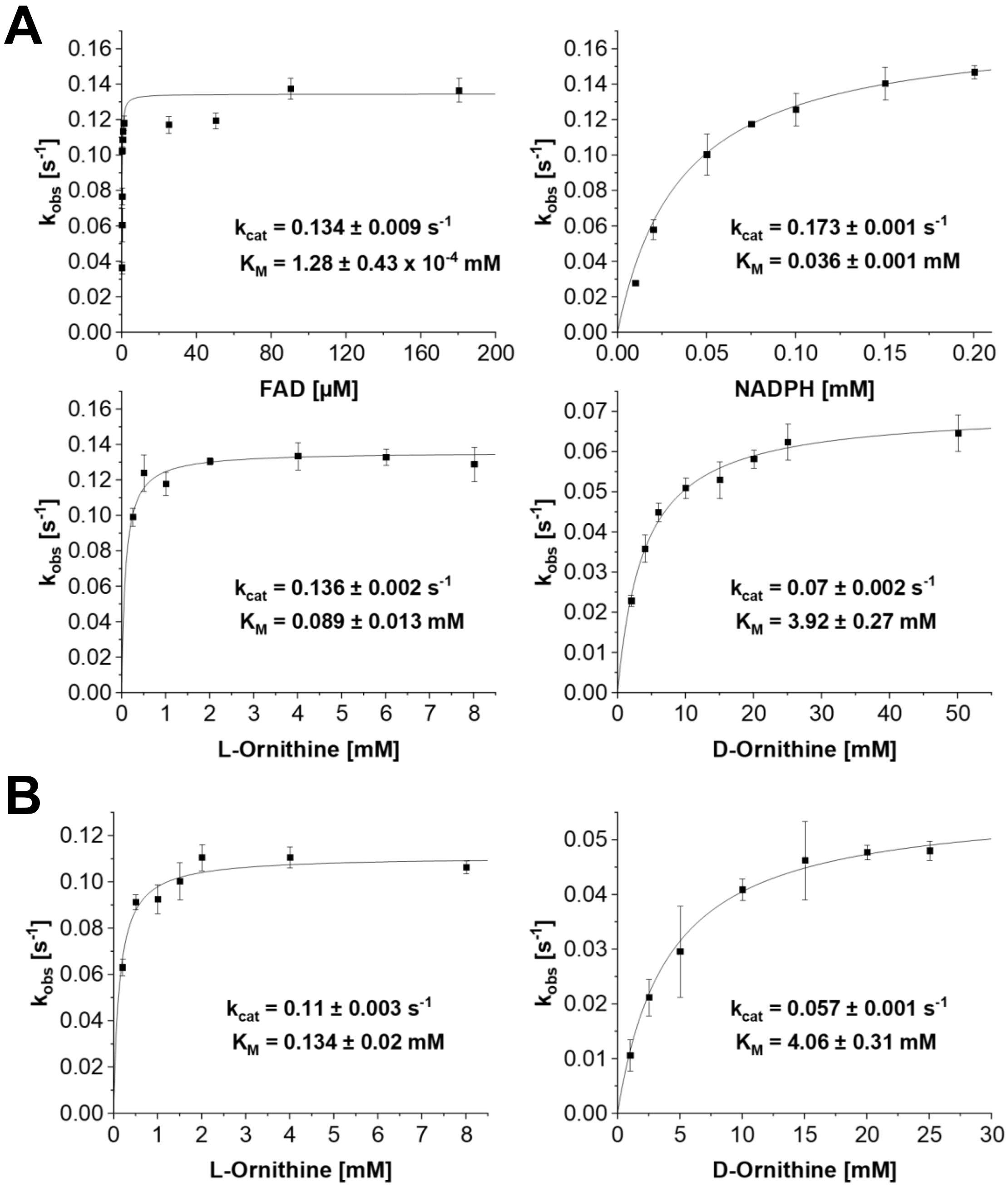

Figure S8*: (A) Kinetic parameters of TheA with varying concentrations of FAD, NADPH, L-Orn and D-Orn measured with the NADPH oxidation assay. (B) Kinetic parameters with varying concentrations of L-Orn and D-Orn measured with the hydroxylation assay.*

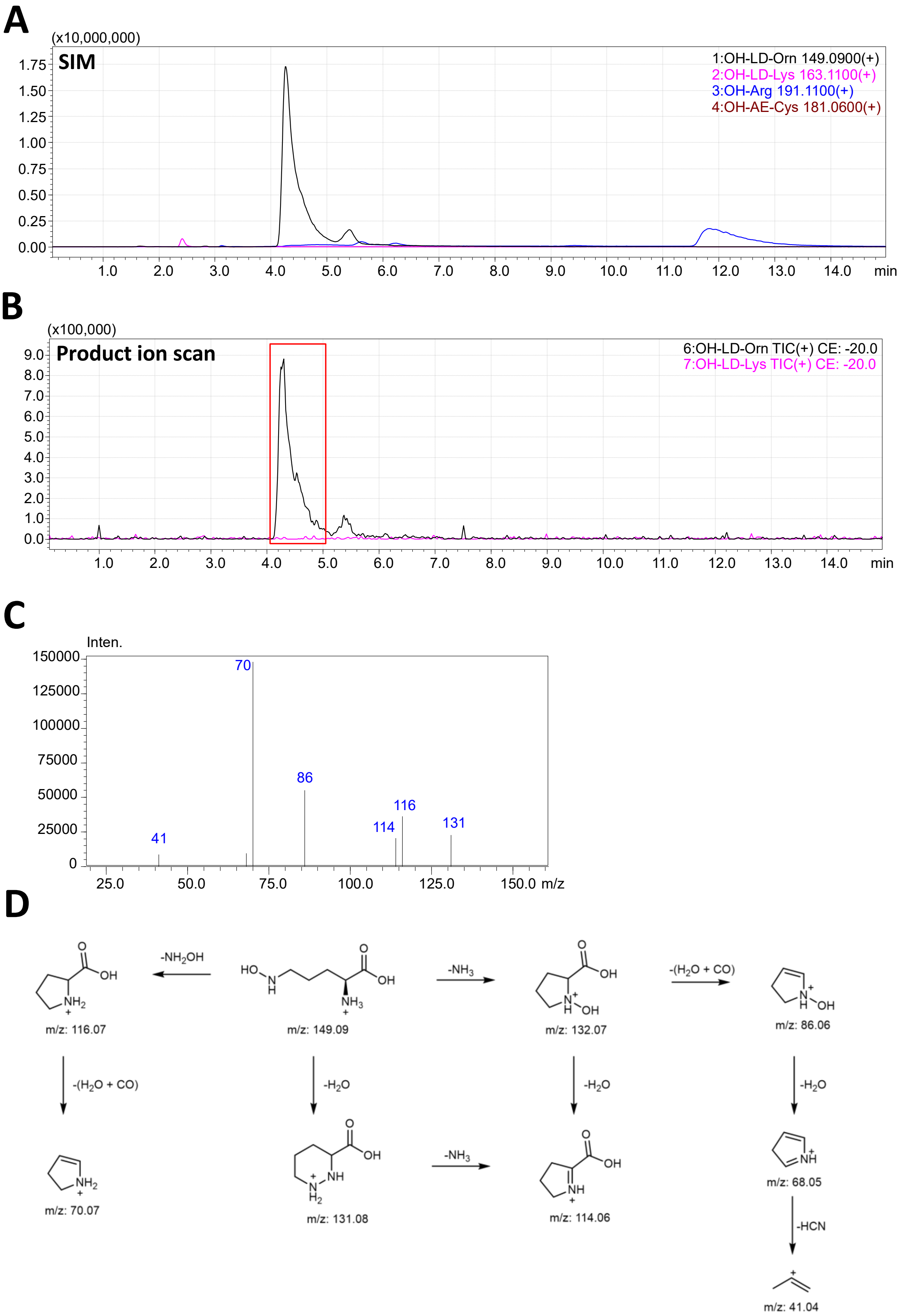

***Figure S9**: LC-MS/MS measurements of TheA biocatalysis with FDH M4, catalase and L-Orn as substrate. (A) Single ion monitoring in positive mode of the expected [M+H]$^+$ ions for the N-hydroxy products. (B) Product ion scan of the potential OH-Orn (precursor m/z (+) = 149.09) and OH-Lys (precursor m/z (+) = 163.11) ions with a collision energy of -20 V. (C) Mass spectrum of the observed product ion scan peak highlighted by the red box. (D) Proposed fragmentation pathway of OH-L-Orn based on previous studies.* [27]

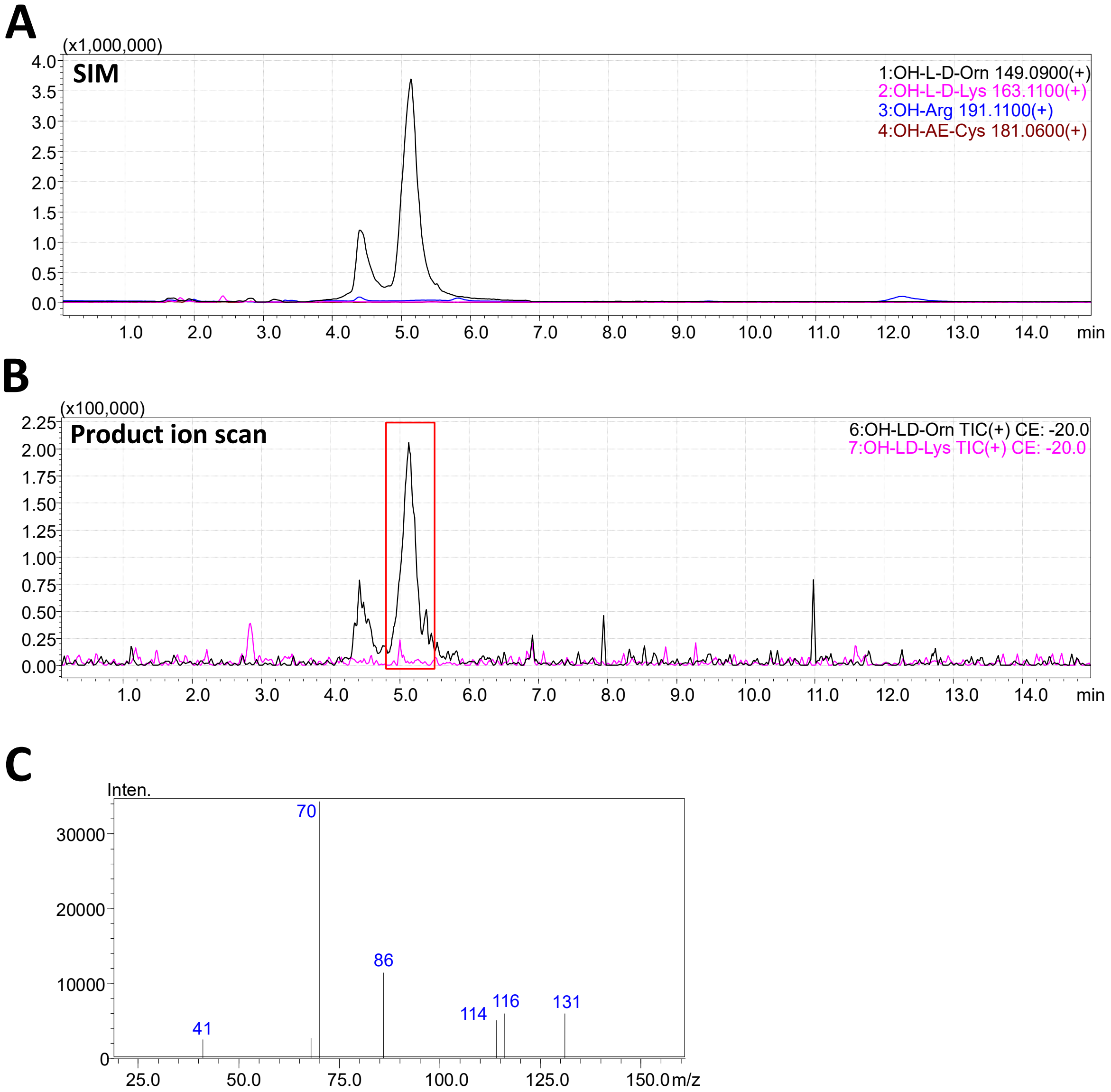

***Figure S10**: LC-MS/MS measurements of TheA biocatalysis with FDH M4, catalase and D-Orn as substrate. (A) Single ion monitoring in positive mode of the expected $[M+H]^+$ ions for the N-hydroxy products. (B) Product ion scan of the potential OH-Orn (precursor m/z (+) = 149.09) and OH-Lys (precursor m/z (+) = 163.11) ions with a collision energy of -20 V. (C) Mass spectrum of the observed product ion scan peak highlighted by the red box.*

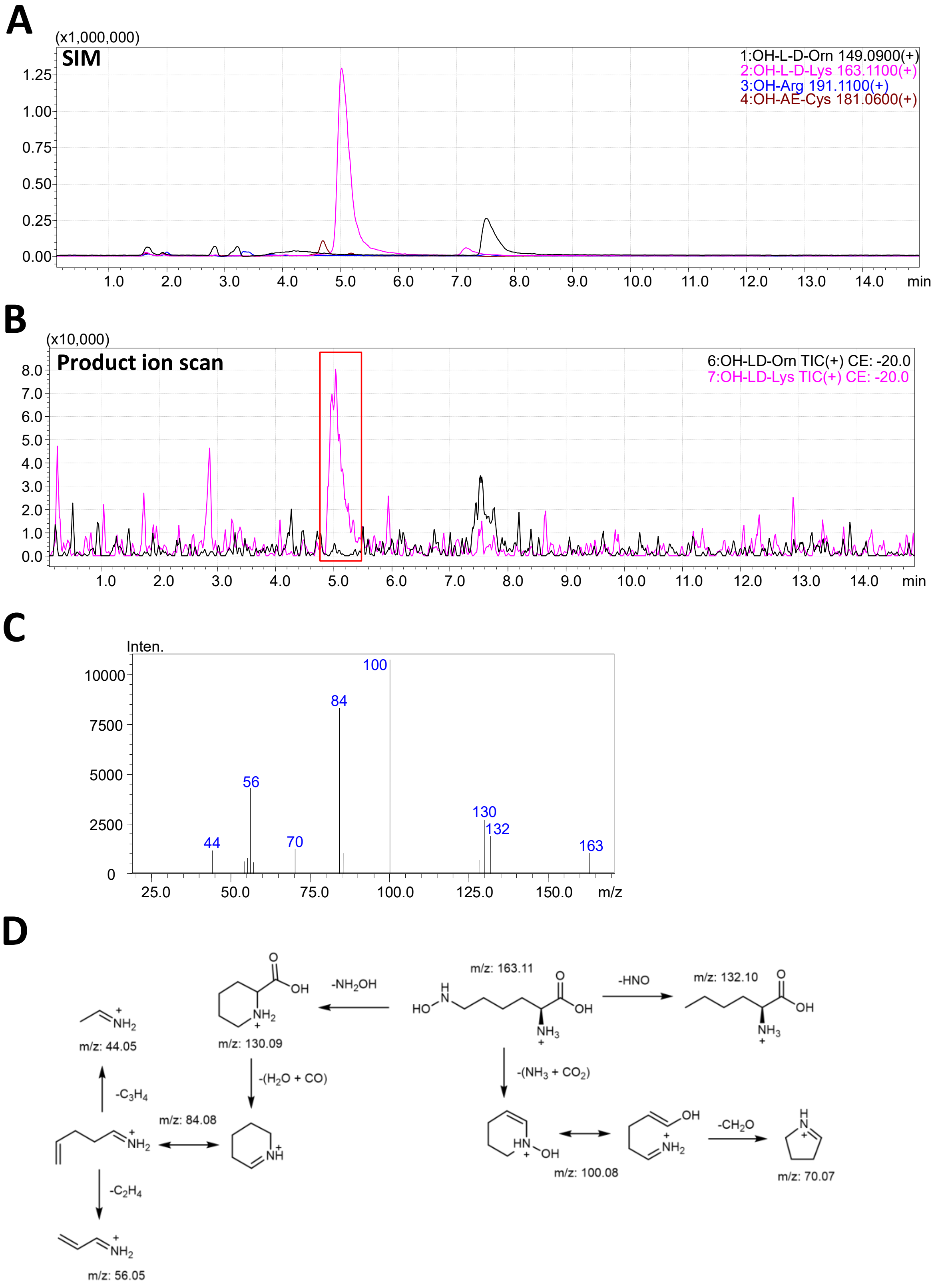

***Figure S11**: LC-MS/MS measurements of TheA biocatalysis with FDH M4, catalase and L-Lys as substrate. (A) Single ion monitoring in positive mode of the expected $[M+H]^+$ ions for the N-hydroxy products. (B) Product ion scan of the potential OH-Orn (precursor m/z (+) = 149.09) and OH-Lys (precursor m/z (+) = 163.11) ions with a collision energy of -20 V. (C) Mass spectrum of the observed product ion scan peak highlighted by the red box. (D) Proposed fragmentation pathway of OH-L-Lys based on previous studies.* [27]

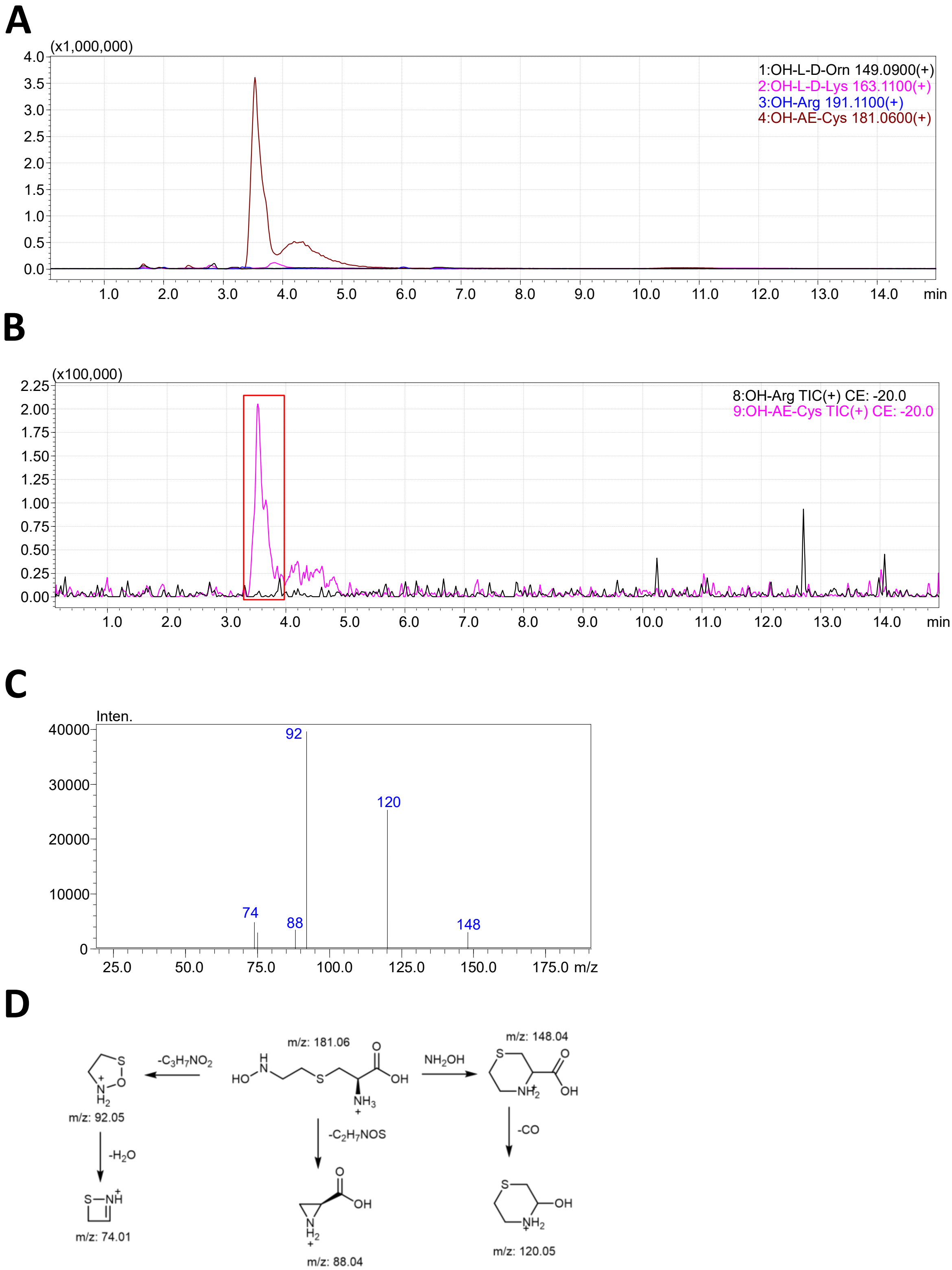

Figure S12: *LC-MS/MS measurements of TheA biocatalysis with FDH M4, catalase and AE-Cys as substrate. (A) Single ion monitoring in positive mode of the expected [M+H]*$^+$ *ions for the N-hydroxy products. (B) Product ion scan of the potential hydroxylated OH-Arg (precursor m/z (+) = 191.11) and OH-AE-Cys (precursor m/z (+) = 181.06) ions with a collision energy of -20 V. (C) Mass spectrum of the observed product ion scan peak highlighted by the red box. (D) Proposed fragmentation pathway of OH-AE-Cys based on previous studies.* [27]

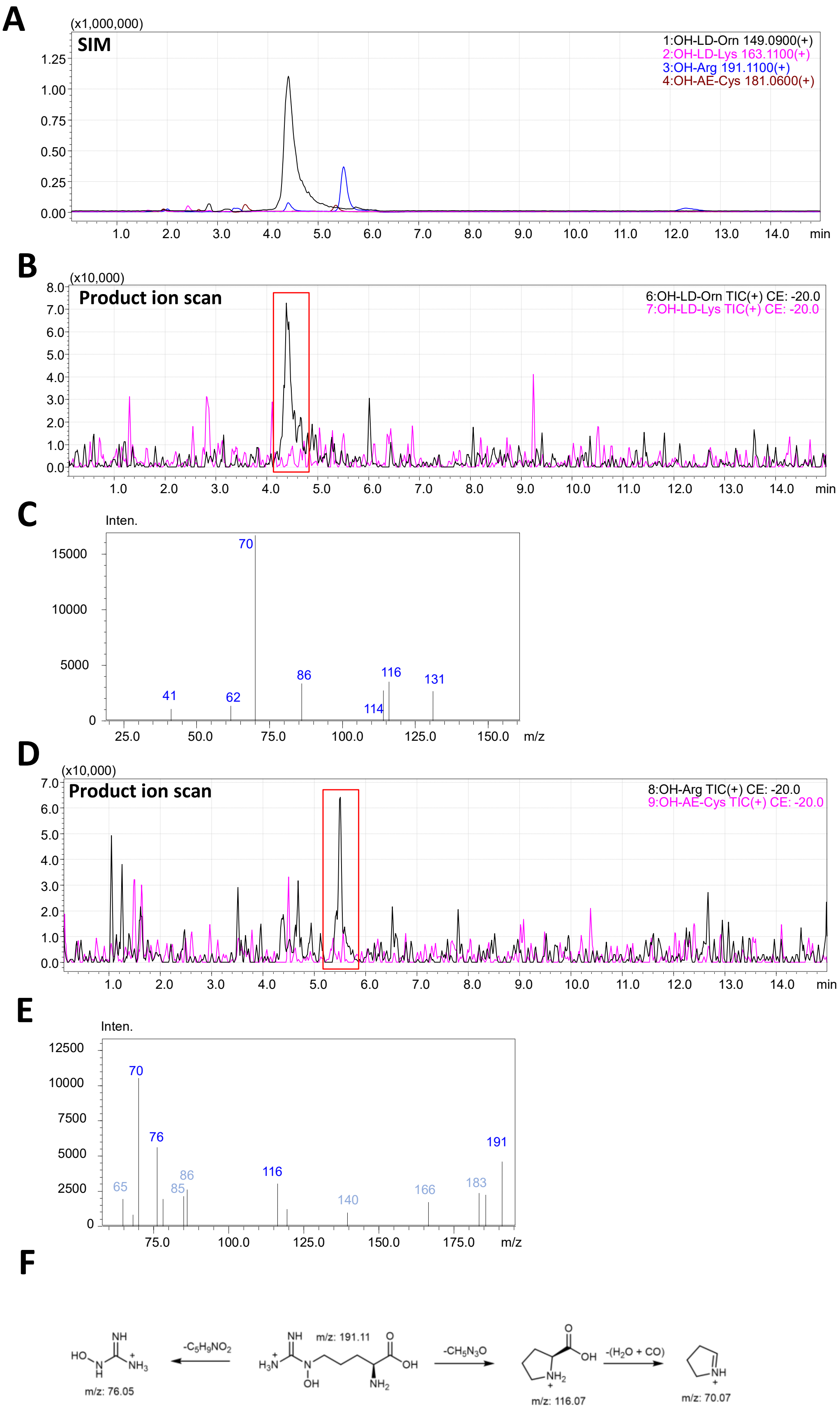

***Figure S13**: LC-MS/MS measurements of TheA biocatalysis with FDH M4, catalase and L-Arg as substrate. (A) Single ion monitoring in positive mode of the expected [M+H]+ ions for the N-hydroxy products. (B) Product ion scan of the potential OH-Orn (precursor m/z (+) = 149.09) and OH-Lys (precursor m/z (+) = 163.11) ions with a collision energy of -20 V. (C) Mass spectrum of the observed product ion scan peak highlighted by the red box. (D) Product ion scan of the potential hydroxylated OH-Arg (precursor m/z (+) = 191.11) and OH-AE-Cys (precursor m/z (+) = 181.06) ions with a collision energy of -20 V. (E) Mass spectrum of the observed product ion scan peak highlighted by the red box. (F) Proposed fragmentation pathway of OH-L-Arg based on previous studies.* [27]

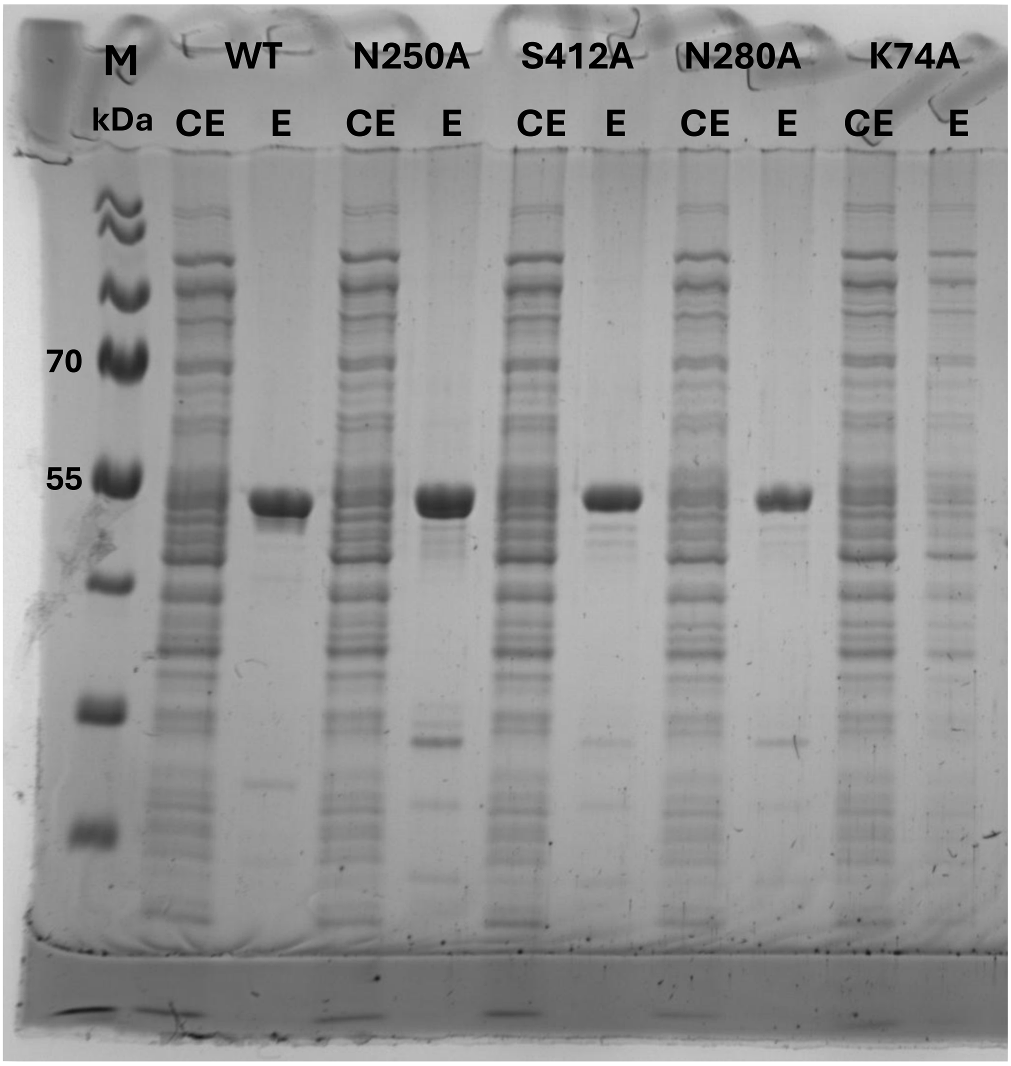

***Figure S14**: SDS-Gel of crude extract (CE) and elution (E) samples acquired during purification of the respective TheA variants.*

[1] C. O. Esuola, O. O. Babalola, T. Heine, R. Schwabe, M. Schlömann, D. Tischler, *J. Mol. Catal., B Enzym.* **2016**, *134*, 378–389.
[2] M. Bosello, A. Mielcarek, T. W. Giessen, M. A. Marahiel, *Biochem.* **2012**, *51*, 3059–3066.
[3] M. Salomone-Stagni, J. D. Bartho, I. Polsinelli, D. Bellini, M. A. Walsh, N. Demitri, S. Benini, *J. Struct. Biol.* **2018**, *202*, 236–249.
[4] R. M. Robinson, P. J. Rodriguez, P. Sobrado, *Arch. Biochem. Biophys.* **2014**, *550-551*, 58–66.
[5] C. A. Madigan, T.-Y. Cheng, E. Layre, D. C. Young, M. J. McConnell, C. A. Debono, J. P. Murry, J.-R. Wei, C. E. Barry, G. M. Rodriguez et al., *PNAS* **2012**, *109*, 1257–1262.
[6] C. Binda, R. M. Robinson, J. S. Del Martin Campo, N. D. Keul, P. J. Rodriguez, H. H. Robinson, A. Mattevi, P. Sobrado, *J. Biol. Chem.* **2015**, *290*, 12676–12688.
[7] A. Thariath, D. Socha, M. A. Valvano, T. Viswanatha, *J. Bacteriol.* **1993**, *175*, 589–596.
[8] J. W. Setser, J. R. Heemstra, C. T. Walsh, C. L. Drennan, *Arch. Biochem. Biophys.* **2014**, *53*, 6063–6077.
[9] L.-A. Giddings, G. T. Lountos, K. W. Kim, M. Brockley, D. Needle, S. Cherry, J. E. Tropea, D. S. Waugh, *PloS one* **2021**, *16*, e0248385.
[10] M. Schrettl, E. Bignell, C. Kragl, C. Joechl, T. Rogers, H. N. Arst, K. Haynes, H. Haas, *World J. Exp. Med.* **2004**, *200*, 1213–1219.
[11] E. E. Smith, E. H. Sims, D. H. Spencer, R. Kaul, M. V. Olson, *J. Bacteriol.* **2005**, *187*, 2138–2147.
[12] T. Heine, M. Mehnert, R. Schwabe, D. Tischler, *SSP* **2017**, *262*, 501–504.
[13] N. R. Saroja, A. H. S. Mohan, D. Srividya, K. Supreetha, *Protein Expr. Purif.* **2019**, *157*, 9–16.
[14] C. Ambrosi, L. Leoni, L. Putignani, N. Orsi, P. Visca, *J. Bacteriol.* **2000**, *182*, 6233–6238.
[15] M. F. Kreutzer, M. Nett, *Org. Biomol. Chem.* **2012**, *10*, 9338–9343.
[16] E. J. Dimise, P. F. Widboom, S. D. Bruner, *PNAS* **2008**, *105*, 15311–15316.
[17] L. Robbel, V. Helmetag, T. A. Knappe, M. A. Marahiel, *Biochem.* **2011**, *50*, 6073–6080.
[18] K. Bufkin, P. Sobrado, *Molecules* **2017**, *22*.
[19] V. Pohlmann, M. A. Marahiel, *Org. Biomol. Chem.* **2008**, *6*, 1843–1848.
[20] J. R. Heemstra, C. T. Walsh, E. S. Sattely, *J. Am. Chem. Soc.* **2009**, *131*, 15317–15329.
[21] H. Y. Kang, S. K. Armstrong, *J. Bacteriol.* **1998**, *180*, 855–861.
[22] H. Y. Kang, T. J. Brickman, F. C. Beaumont, S. K. Armstrong, *J. Bacteriol.* **1996**, *178*, 4877–4884.
[23] D. Lynch, J. O'Brien, T. Welch, P. Clarke, P. O. Cuív, J. H. Crosa, M. O'Connell, *J. Bacteriol.* **2001**, *183*, 2576–2585.
[24] K. Tamura, G. Stecher, S. Kumar, *Mol. Biol. Evol.* **2021**, *38*, 3022–3027.
[25] R. C. Edgar, *Nucleic Acids Res.* **2004**, *32*, 1792–1797.
[26] a) J. Jumper, R. Evans, A. Pritzel, T. Green, M. Figurnov, O. Ronneberger, K. Tunyasuvunakool, R. Bates, A. Žídek, A. Potapenko et al., *Nature* **2021**, *596*, 583–589; b) H. Land, M. S. Humble, *Methods Mol. Biol.* **2018**, *1685*, 43–67;
[27] P. Zhang, W. Chan, I. L. Ang, R. Wei, M. M. T. Lam, K. M. K. Lei, T. C. W. Poon, *Sci. Rep.* **2019**, *9*, 6453.
